# Do ethics matter? Impact of ethics on organizational citizenship behavior: a meta-analysis

**DOI:** 10.3389/fpsyg.2026.1773853

**Published:** 2026-05-19

**Authors:** Zhihong Li, Xinya Yang, Jun Tang

**Affiliations:** 1 School of Public Administration and Policy, Renmin University of China, Beijing, China; 2 Faculty of Humanities Social Sciences, Nanjing Forestry University, Nanjing, China

**Keywords:** ethics, OCB, MASEM, boundaries, dual and multi-paths, impact mechanisms

## Abstract

Ethical constructs have been identified as a direct, effective, and economical way to influence organizational citizenship behavior (OCB). Although numerous studies have demonstrated the critical role of ethical factors in inhibiting or promoting OCB, a comprehensive quantitative synthesis remains lacking. Therefore, we conducted a meta-analysis to quantitatively synthesize 125 independent studies (28 ethical factors, 331 effect values, and 158,336 participants) and propose a theoretical framework for the relationship between ethics and OCB. The results revealed that 24 ethics factors were significantly and positively related to OCB, whereas moral disengagement was significantly and negatively associated with OCB. Furthermore, we found that ethical responsibility is indirectly associated with OCB through multiple pathways. Specifically, it is positively linked to OCB through ethical leadership and ethical climate (e.g., paths CSR → EC → OCB, CSR → EL → OCB, CSR → EC → MI → OCB, CSR → EL → EC → OCB, and CSR → EL → EC → MI → OCB). Conversely, it shows a negative link to OCB through ethical identity (e.g., paths CSR → MI → OCB and CSR → EL → MI → OCB). Our analysis highlights the important connection between ethical factors and OCB and provides suggestions for future research.

## 1 Introduction

Despite the increasing prominence of business ethics, the specific mechanisms underlying the link between ethical constructs and organizational citizenship behavior (OCB) remain insufficiently underexplored (Liao et al., 2026; Orlitzky et al., 2003; Shahzad et al., 2025). OCB is characterized by discretionary actions that transcend established job responsibilities and formal compensation agreements, while significantly enhancing organizational effectiveness (Organ, 1997). Given that OCB comprises voluntary and autonomous actions that is beneficial to others or the organization, it is, by its nature, a value-based phenomenon (Organ, 1997). The decision to engage in or refrain from such behaviors is fundamentally a moral choice (Wang and Sung, 2014). Consequently, analyzing OCB from an ethical perspective is essential for understanding how ethical contexts shape employees’ discretionary behaviors.

In recent years, high-profile corporate scandals have significantly heightened public scrutiny regarding organizational ethics, prompting companies to institutionalize ethical standards within their daily practices (Chen et al., 2016; Wang et al., 2025). However, a fundamental tension persists within the corporate landscape: prioritizing ethical operations may sometimes conflict with the traditional imperative of profit maximization (Wang and Sung, 2014). Consequently, scholars in the field of management have increasingly explored the impact of ethical factors on employees’ behaviors across multiple dimensions, including OCB, examining constructs such as ethical climate (Zhang and Zhu, 2020), ethical culture (Ruiz-Palomino and Martínez-Baños, 2014), ethical leadership (Wang and Sung, 2014), and moral identity (Mansur et al., 2020). Although the literature on this topic continues to expand, empirical findings remain fragmented and inconclusive. For example, while numerous studies indicate a positive association between ethical climate and OCB (e.g., Deng, 2020; Oh, 2022; Yu and Chen, 2015), other investigations have reported negative or non-significant correlations (such as Liu, 2012; Hung and Tsai, 2015).

To date, comprehensive literature reviews and meta-analyses addressing this topic remain limited. Existing studies have predominantly focused on single concepts, such as ethical leadership within specific domains, or have adopted predominantly theoretical orientations toward ethics. However, without providing a quantitative mapping of the underlying mechanisms (e.g., Caldwell, 2011; Ogunfowora et al., 2022; Wang and Wang, 2018). While a recent systematic review has highlighted the plausible pathways linking integrity, OCB, and broader management outcomes (Saputra et al., 2026), a comprehensive quantitative meta-analysis mapping the precise magnitude of these relationships across diverse ethical domains is still lacking. This gap can be addressed through a quantitative synthesis. Meta-analytic techniques serve as a powerful methodological remedy that corrects for statistical artifacts and biases attributable to sampling error and study design, thereby establishing cumulative and reliable empirical evidence regarding the true strength of these relationships (Anonymous, 2021; Gong et al., 2026; Hunter and Schmidt, 2004; Li et al., 2020; Tang and Li, 2026). It is essential to precisely identify the factors that influence OCB to enable researchers and managers to accurately assess the antecedents propelling employees’ positive actions.

Accordingly, this study aims to synthesize the fragmented literature by organizing the broad umbrella of “ethics” into three distinct conceptual boundaries (or domains): (1) individual moral traits and cognitions (e.g., moral identity, moral reasoning), (2) interpersonal and leadership dynamics (e.g., ethical leadership), and (3) macro-level organizational contexts (e.g., ethical climate, ethical responsibility (CSR). By delineating these boundaries, we provide a more theoretically disciplined mapping of how different ethical domains relate to OCB. First, we develop a theoretical model and propose hypotheses regarding these associations. Second, we conduct a meta-analysis utilizing a random-effects model to determine not only which ethical factors influence OCB but also the magnitude of their effects. Third, we employ meta-analytic structural equation modeling (MASEM) to examine the multiple mediating pathways linking CSR to OCB, thereby delineating a complex structural path. Finally, we explore the theoretical and practical implications of our findings and delineate avenues for future research.

Through these efforts, this study makes three primary contributions to the literature on ethics and organizational behavior. First, we offer the broadest quantitative synthesis to date of the link between ethics and OCB. By integrating 125 studies across 28 distinct ethical constructs, we reconcile previous theoretical inconsistencies and elucidate the true magnitudes and directions of these effects. Second, we expand the nomological network of OCB through the systematic identification not only of the ethical drivers that promote OCB (e.g., ethical leadership, ethical climate) but also of key inhibitors such as moral disengagement. Third, we theoretically advance the literature by using MASEM to explore how macro-level ethics are associated with micro-level behaviors. Specifically, by analyzing multiple mediating pathways, we demonstrate that CSR is positively linked to OCB via structural pathways such as ethical leadership and climate; concurrently, we uncover a subtle but potentially inhibitory pathway involving moral identity. Ultimately, rather than merely asserting that ethics matter, this study goes beyond prior work by elucidating the specific mechanisms through which ethical contexts shape employee behavior.

## 2 Theoretical background and hypothesis development

### 2.1 Conceptualization of organizational citizenship behavior

The theoretical foundation of OCB can be traced back to Barnard’s (1968) notion that the spontaneous willingness of individuals to work together is essential to bridge gaps within formal organizational structures (Barnard, 1968). Organ (1988) subsequently defined OCB as a form of discretionary work behavior that is (1) extra-role (not formally required), (2) unrewarded by formal compensation systems, and (3) beneficial to the broader organization (Organ, 1988).

**Figure 1:**
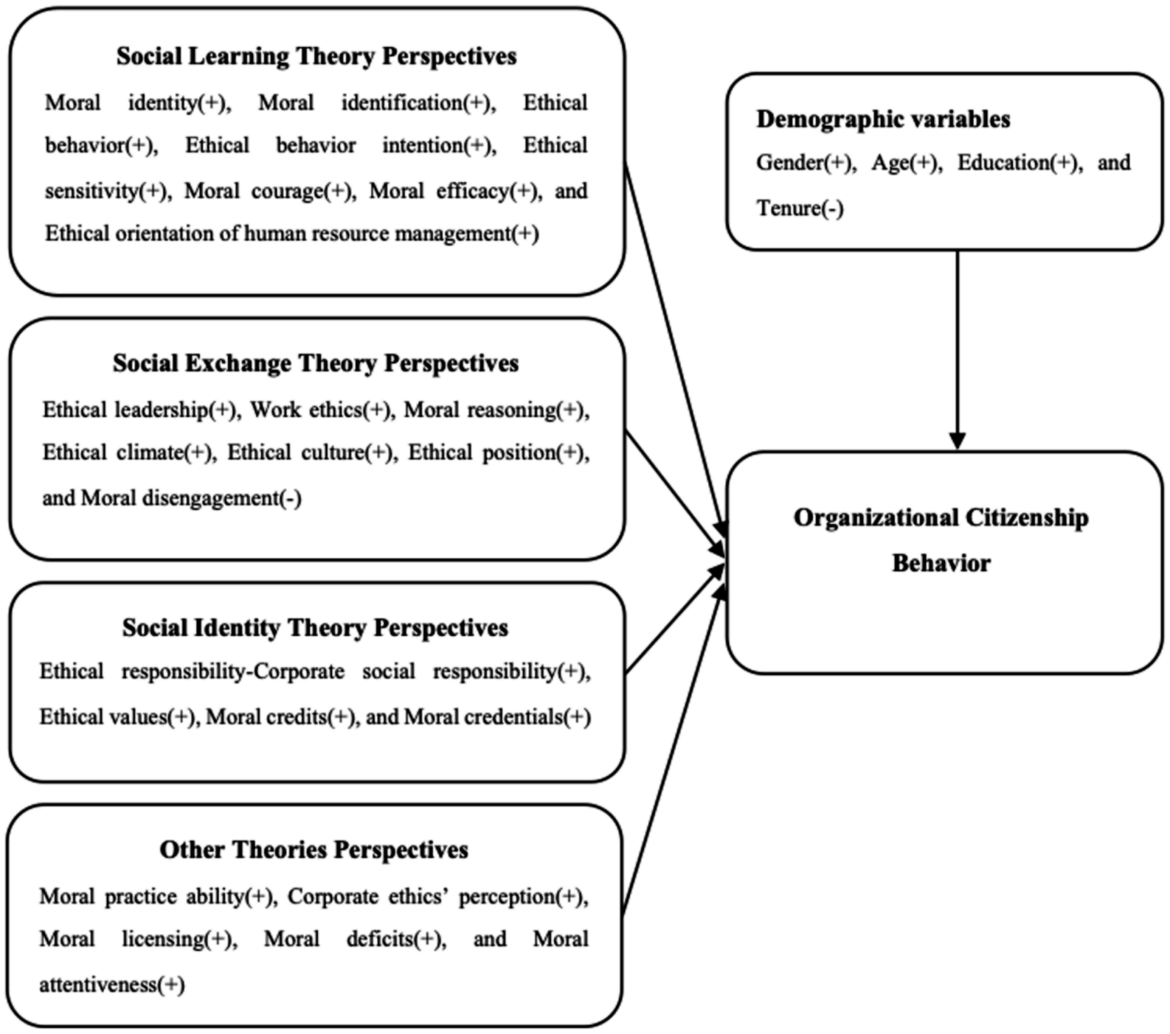
Conceptual model.

Prior research typically bifurcates OCB into two distinct dimensions: individual-oriented (OCB-I) and organization-oriented (OCB-O) (Podsakoff et al., 2009; Williams and Anderson, 1991). OCB-I consists of interpersonal behaviors like altruism, helping and personal assistance that aid colleagues and, consequently, indirectly improve the organization (Lee and Allen, 2002). Conversely, OCB-O refers to behaviors directed toward the organization as a whole, including civic virtue, sportsmanship, and organizational compliance (Williams and Anderson, 1991). Because OCB is inherently voluntary and prosocial, engaging in it represents a value-based, ethical decision. To systematically unravel the mechanisms by which various ethical factors facilitate or inhibit these behaviors, we utilize Social Learning Theory, Social Exchange Theory, and Social Identity Theory as an organizing heuristic (see Figure 1). Rather than serving as a mere classification scheme, this theoretical integration is analytically necessary to understand how different ethical domains motivate citizenship behavior. These theories are not deployed as mutually exclusive silos, but rather as analytical lenses to delineate the functional pathways of different ethical domains. Specifically, SLT is utilized to explicate the observational modeling of ethics (e.g., how individual traits and leadership behaviors are emulated); SET is applied to analyze reciprocal workplace dynamics (e.g., how ethical climates and work ethics dictate socioemotional exchange norms); and SIT is employed to explain the internalization of macro-level organizational prestige (e.g., how corporate social responsibility shapes employee identification).

#### 2.1.1 Social learning theory (SLT)

Based on Social Learning Theory (Bandura, 1977), individuals learn a new behavior through observation of and imitation from other people’s actions in the society. Leaders and those in positions of authority are primarily the references for appropriate behavior at work. Organization’s agents’ ethics, responsibilities and services have formed the normative Standards of behavior for employees within organizations. The observation of this kind of behavior helps motivate others to exhibit similar positive behaviors, i.e., OCB. By setting a moral example, organizations enhance employees’ ethical cognition and foster their intrinsic drive to emulate pro-environmental, knowledge-sharing, and helpful behaviors. Consequently, ethical factors that encapsulate an individual’s moral cognitive frameworks and modeling mechanisms serve as critical drivers of OCB (Zhang and Zhu, 2020; Shahzad et al., 2025). Therefore, we hypothesize the following:

Hypothesis 1: Moral identity (a), moral identification (b), ethical behavior (c), ethical behavior intention (d), ethical sensitivity (e), moral courage (f), moral efficacy (g), and ethical orientation of human resource management (h) are positively related to OCB.

#### 2.1.2 Social exchange theory (SET)

Social Exchange Theory (Blau, 1964) provides a robust framework for understanding the reciprocal relationships between employees, their leaders, and the organization. At its core, SET is predicated upon the norm of reciprocity: when individuals receive socioemotional or economic benefits from an exchange partner, they feel an obligation to reciprocate. Ethical leaders and ethical climate facilitate the formation of high-quality, supportive social exchange relationships. Recent empirical models in public-sector contexts reinforce this by conceptualizing integrity as an organizational ethical infrastructure; when leadership, organizational policy, and a supportive work environment are established, they directly relate to OCB through this underlying integrity mechanism (Syamsir et al., 2026). When organizations treat employees equitably, provide them with development opportunities, and cultivate a nurturing environment, these employees will develop a sense of obligation. Employees fulfill their responsibilities by demonstrating greater organizational loyalty, job satisfaction, and engaging in more OCB. Ethical violations and moral disengagement disrupt reciprocal behavioral norms, thereby reducing pro-organizational actions (Den Hartog and Belschak, 2012; Ogunfowora et al., 2022). Therefore, we hypothesize the following:

Hypothesis 2: Ethical leadership (a), work ethics (b), moral reasoning (c), ethical climate (d), ethical culture (e), and ethical position (f) are positively associated with OCB, whereas moral disengagement (g) is negatively related.

#### 2.1.3 Social identity theory (SIT)

Social Identity Theory (Ashforth and Mael, 1989; Ellemers et al., 2004) posits that an individual’s self-concept is partially contingent upon their membership in social groups, such as the organizations in which they are employed. When individuals experience a high level of identification with an organization, they internalize the organization’s norms and take pride in the group’s reputation, thereby being more likely to engage in OCB. Organizations that have set up ethical standards in conducting corporate social responsibility will improve their company’s public image and inner moral awareness externally. Thus, enhancing the employees’ trustworthiness and belonging to the organization (Shahzad et al., 2025; Wang and Wang, 2018). Ethical responsibility usually means the duties owed by an enterprise to different parties such as society at large, nature, employees, consumers, governments and other subjects of interests (Turker, 2009a; Turker, 2009b).

However, it is recognized that ethical responsibility may generate two types of responses for OCB. Firstly, authentic ethical responsibility innovations (such as environmental protection, fairness inclusiveness and charity activities) create an ethical environment and set examples for ethical behavior that motivate OCB (Maignan and Ferrell, 2004). On the one hand, if employees believe that such ethical duties are not sincere but instead merely an “impostor white washing”, then they may develop negative feelings towards them. This disillusionment diminishes the employees’ moral identity and identification with the organization, activating an inhibitory pathway that ultimately reduces OCB (Gond et al., 2017; Monin and Miller, 2001; Shahzad et al., 2025). Therefore, we hypothesize the following:

Hypothesis 3a: Ethical responsibility is positively related to OCB.

Hypothesis 3b: Ethical responsibility positively influences OCB through the mediating effects of ethical leadership and ethical climate.

Hypothesis 3c: Ethical responsibility negatively influences OCB through the mediating effect of moral identity.

Consistent with the tenets of SIT, an organization’s embedded moral standing further reinforces an employee’s organizational identification. Factors that signal the organization’s moral legitimacy similarly drive pro-organizational behavior (Gond et al., 2017; Shahzad et al., 2025), such as ethical values, moral credits, and moral credentials. Therefore, we hypothesized the following:

Hypothesis 4: Ethical values (a), moral credits (b), and moral credentials (c) are positively related to OCB.

#### 2.1.4 Other theories perspectives

Through previous research, we have found that these factors can also affect OCB, such as moral practice ability, corporate ethics perception, moral licensing, moral deficits, and moral attentiveness. Therefore, we hypothesized the following:

Hypothesis 5: Moral practice ability (a), corporate ethics perception (b), moral licensing (c), moral deficits (d), and moral attentiveness (e) are positively related to OCB.

### 2.2 Demographic factors on OCB

Although ethical constructs and organizational climate serve as primary drivers of organizational citizenship behavior (OCB), employees’ demographic characteristics specifically gender, age, education level, and organizational tenure also fundamentally shape their propensity to engage in discretionary behaviors.

Drawing on social role theory, gender socialization influences relational and communal behaviors in the workplace. Differing gender role expectations often shape how employees perceive and perform interpersonal helping (OCB-I) and civic virtue (OCB-O) (Estiri et al., 2018; Hackett et al., 2018). Socioemotional selectivity theory posits that with age, individuals tend to move from a focus on instrumental career advancement to one of socio-emotional connection and generativity. As a result, the older workers have greater organizational commitment and willingness to assist others; therefore, these behaviors fall under OCB. In terms of education, according to human capital theory and role theory, workers who are educated more highly have greater cognitive abilities and a deeper understanding of the organization. Those employees who are well-educated often have a higher sense of role-breadth self-efficacy, i.e., they believe in being able to handle additional responsibilities beyond the scope of their work assignments. In addition, higher education tends to cultivate stronger professional norms of reciprocity and public spirit among them that make them more inclined towards OCB expectations. Finally, organizational tenure also exhibits complex relationships with OCB. Drawing on psychological contract theory and resource depletion models, while early-tenure employees may frequently engage in OCB to build social capital and establish their reputation, extended tenure often leads to a shift in these dynamics (Kim, 2018). Over time, long-tenured employees may experience role overload or begin to view previously voluntary extra-role behaviors as expected in-role duties, thereby reducing the frequency of discretionary OCB. Therefore, we hypothesized the following:

Hypothesis 6: Gender (a), age (b), and education (c) are positively related to OCB; and tenure (d) negatively related with OCB.

## 3 Method

### 3.1 Literature search

**Figure 2:**
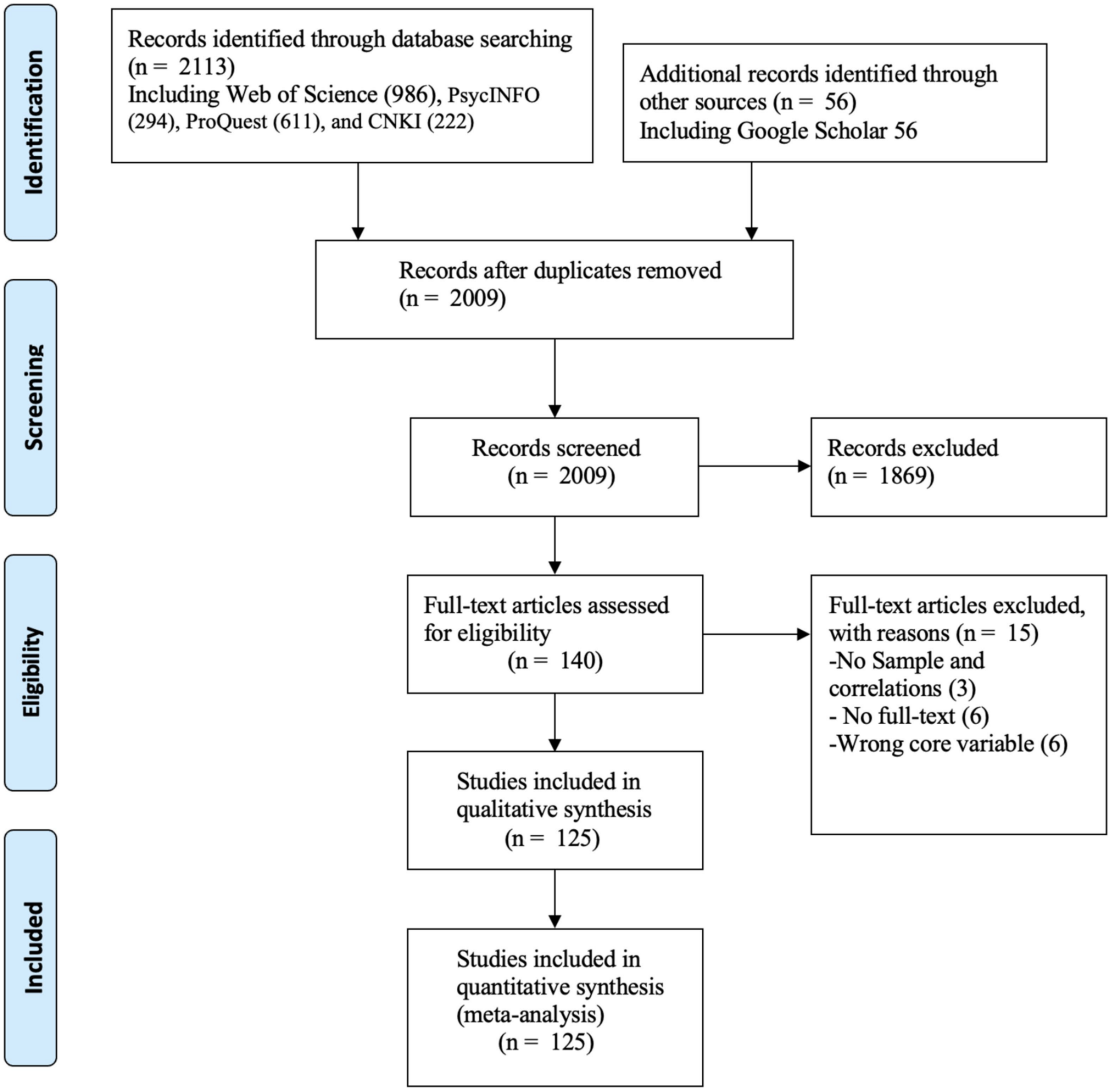
PRISMA searching flow diagram.

A systematic literature search was conducted in consultation with a research librarian to identify relevant published and unpublished studies. First, we searched the following international electronic databases: Web of Science, PsycINFO, ProQuest, CNKI (China National Knowledge Infrastructure), and Google Scholar (capturing literature published up to October 2, 2025) (Barends and Rousseau, 2018; Li et al., 2020; Tang and Li, 2026). The systematic search was performed by combining each of the possible combinations of three sets of keywords: (1) ethics-related keywords (e.g., “ethic* leadership,” “ethic* climate*,” “ethic* value*,” “ethic* responsibility,” “ethic* behavior*”); (2) ethics constructs (e.g., “moral ident*,” “moral behavior*,” “moral credit*,” “moral leadership,” “moral reasoning,” “moral justice,” “ethical responsibility”); and (3) organizational citizenship behaviors (e.g., “organization* citizenship behavior*”, “OCB*”, “help* behavior*”, “sportsmanship”, “self-development”, “voice”, and “civic virtue”), a strategy consistent with previous meta-analyses (e.g., Organ et al., 2006; Podsakoff et al., 2009). Second, in order to minimize the impact of publication bias and comprehensively capture potentially relevant literature, we also included unpublished conference papers, research plans, and theses in the aforementioned databases. Finally, the snowball method was used to review and consult the reference sections of the retrieved articles to identify additional potential sources. Overall, our initial search yielded 2,169 studies for eligibility screening. The search process is illustrated in Figure 2.

### 3.2 Inclusion criteria

The following four criteria were used to determine whether studies were included in the meta-analysis: First, studies had to empirically examine the associations of interest (ethics to OCB) of interest. Second, the study had to report sufficient data to calculate the effect size (e.g., Pearson’s *r*, Cohen’s *d*, and sample size) for at least one focal relationship under investigation. Third, to ensure applicability to the business and management domain, we only included preliminary research conducted within organizational contexts, such as samples from employees and managers. Fourth, considering the potential differences in research designs and variable measurement methods, we only included studies that measured ethical factors and organizational citizenship behavior using self-report instruments. Because ethical factors are essentially intrinsic and subjective, this makes self-reporting highly relevant and the best obtainable method. Admittedly, this design choice increases the risk of same-source inflation and common-method bias. However, this parameter was analytically necessary because most of the core constructs synthesized in this review—such as moral identity, moral reasoning, and ethical intentions—are fundamentally internal psychological states that are most accurately captured via self-reports. To mitigate the risks, we added additional sensitivity analysis to address this potential inflation in our robustness checks and interpreted highly correlated constructs with caution.

### 3.3 Coding process

To ensure coding reliability, we set up a coding group composed of three researchers with knowledge and methodological experience in business and management. Each included study was independently coded by two researchers. First, the two researchers reached an agreement on coding standards through frequent discussions in the coding group and began the first round of experimental coding, covering multiple research features pertinent to later analysis. These included author, publication year, region, sample size, industry type, demographic characteristics (gender, age, and education level), ethical factors, and types of organizational citizenship behavior. Second, the two coders independently encoded all relevant research features from all the studies based on the revised coding scheme. Third, the two coders cross-checked and verified that the coder’s code consistency reached 95% and then merged the results into the final code table. During the coding process, any issues encountered were resolved by the team, and through collective deliberation to finalize the final coding table.

### 3.4 Moderating effect analysis

In meta-analysis, there are many independent empirical studies, and similarity is a prerequisite for conducting meta-analysis. Weighted merging can be used to handle synthesize statistical information. Through the Q value analysis of the 125 empirical studies, it was greater than the corresponding Chi-square value, which indicates that the effect sizes of the samples used in this study are heterogeneous. In addition, the values of I^2^ were mostly higher than 75% (considered high heterogeneity in meta-analysis standards). For example, one value of *I*^2^ is 96.06%, indicating that the difference of actual effect size accounts for 96.06% of the observed change. Random error accounts for only 3.94% of the observed variance. Therefore, it can be concluded that there are moderating variables in the selected sample literature that affect the correlation between ethical factors and OCB. Many potential factors may lead to inconsistencies in previous investigations of ethical factors and organizational citizenship behavior. Based on prior literature, we identified publication year, gender ratio, cultural background (such as national development level in different geographical regions), sample population types, funding support and other factors as potential moderators (Hunter and Schmidt, 2004; Li et al., 2020; Mayer et al., 2012; Tang and Li, 2026). To conduct our moderating effect analyses, we established a minimum threshold of *k* ≥ 3 for each subgroup to ensure statistical stability. We explicitly operationalized our moderators as follows: (1) Publication year was treated as a continuous variable; (2) Cultural background (national development level) was categorized into “developed” versus “developing” economies based on the United Nations Country Classifications to capture macro-economic and cultural differences; (3) Sample population was strictly divided by male% (“<49%”, ‘49%–51%”, “>51%”); and participant samples were categorized into “employees”, “managers”, “mixed”, and “students”; and (4) Funding was coded as “funded” (explicitly reporting support from national, university, or institutional grants) versus “unfunded” (no grant support reported). By clearly defining these boundaries, we aimed to unpack the high heterogeneity (*I*^2^ > 75%) observed in the main effects.

### 3.5 Meta-analytic procedures

In order to generate robust estimates of the focal relationships, we used Hunter and Schmidt’s (2004) random effects model for the analysis; Microsoft’s Excel 2021 (version 16.54) to extract and organize the data; R (version 4.2.3)’s “psychmeta” and “metaSEM” packages alongside Mplus software [Mplus 8, version 1.6(1)] to perform meta-analysis, multiple-chain mediated models’ analysis, and publication bias analysis, etc. First, the correlation coefficients reported in each study and their internal consistency reliability coefficients were used to calculate the true correlation coefficient (*ρ*) between ethical factors and OCB (ρ also known as the zero-order correlation coefficient, which is the correlation coefficient after the weighted observation of the average sample size or the real correlation coefficient after measurement error correction). For a few studies that reported other effect sizes (such as *d*, mean and standard deviation), we converted them into a unified comprehensive effect size Pearson’s r. For studies that did not report reliability, internal consistency reliabilities tend to overestimate the reliability of score-based criteria (and, in turn, underestimate corrected correlations), *α* corrects for attenuated effect sizes because internal measurement error is typically smaller than inter-measurement error (LeBreton and Senter, 2008). Therefore, we imputed the weighted average of all studies reporting the reliability of this variable. For studies that included multiple measures and/or outcome measures, we calculated a composite r prior to inclusion in the overall meta-analysis (Schmidt and Le, 2004). Next, to better assess the impact of the publication bias profile of the included studies, we used a combination of Fail-safe N (Rosenthal) and Egger regression because both methods have distinct advantages and disadvantages for testing different samples. For example, Egger regression is prone to one type of error for small sample sizes and, therefore, Egger regression is not applicable to small Fail-safe N (Rosenthal). We compensated for this shortcoming by reflecting the required number of studies to reverse the results of the meta-analysis. We allowed for estimating how the number of studies with potentially unpublished negative results, affected the results of the positive meta-analysis (Li et al., 2020; McDaniel et al., 2006; Tang and Li, 2026). It is difficult to shift the results of a meta-analysis when Fail-safe N is much larger than the number of included studies, especially when the number of required studies exceeds 5 K + 10, where K is the number of included studies (Card, 2011). However, given that their judgment criteria are not the golden rule, we combined them to comprehensively assess the impact of the included studies owing to publication bias. Next, we used the MASEM technique to test a dual and pathway mediation model consisting of ethical responsibility, ethical leadership, ethical climate, moral identity, and OCB. This technique could test our hypothesized theoretical model and explain the relationship between variables or compare multiple models supported by theory to test the strengths and weaknesses of the models (Bilgili et al., 2017). First, we created a meta-analytic correlation matrix for these five variables by extracting the correlation coefficients, confidence intervals, and sample sizes between these variables in the included studies and then standardized the sample sizes in MASEM by calculating the harmonic mean to address the effects of using different sample sizes. The harmonic mean (also known as the inverse mean) is the inverse of the arithmetic mean of the overall statistical variables, (which is a more conservative estimate than the arithmetic means) and balances the effect of large values relative to small values (Bilgili et al., 2017; Viswesvaran and Ones, 1995). Finally, we used the maximum likelihood estimation method and performed the MASEM analysis.

### 3.6 Robustness checks

Given the substantial heterogeneity observed (*I*^2^ > 75%), we conducted rigorous robustness checks to substantiate the validity of our findings. Specifically, we performed leave-one-out sensitivity analyses (influence diagnostics) for the main effects to verify that no single study disproportionately drove the meta-analytic estimates. The overall effect sizes and significance levels remained robust. Furthermore, we conducted specific sensitivity tests for constructs with a small number of studies (*k* < 5). While the direction of these effects remained consistent, the magnitude of point estimates for these small *k* constructs showed greater volatility, indicating that future primary research is warranted to stabilize these specific estimates.

## 4 Results

The results of the meta-analysis evaluating the relationships between ethical factors and OCB are shown in Table 1. Overall, of across the 28 ethical factors examined, 24 were significantly correlated with OCBs, with the vast majority having true effect values of 0.30 or higher. Thus, ethics has a significant and substantive associations (i.e., moderate to strong) with OCB.

**Table 1:** Meta-analysis results for ethical factors and OCB.

Ethical factors	*𝑘*	*N*	*𝑟*	*𝑺𝑫𝑟*	*𝛒*	*𝑆𝐷𝝆*	95% CI		80 %CV		Q (*p* value)	*I^2^*	Fail-safe N (Rosenthal)	Egger’s test	
														t	*p* value
*Moral identity-OCB*	24	7,588	0.42	0.20	0.51	0.22	0.41	0.61	0.25	0.77	331.11***	93.05	7,153	2.45	0.02
Moral identity-OCBI	9	2,961	0.43	0.26	0.51	0.28	0.31	0.70	0.18	0.84	186.29***	95.71	1,022	1.11	0.30
Moral identity-OCBO	5	1,689	0.43	0.18	0.54	0.19	0.27	0.81	0.31	0.77	52.62***	92.40	306	4.89	0.02
*Moral identity-Internalization-OCB*	3	1,368	0.41	0.26	0.49	0.29	0.05	0.93	0.16	0.82	92.79**	97.84	178	0.67	0.63
Moral identity-Internalization-OCBI	1	466	0.65		0.73										
Moral identity-Internalization-OCBO	1	466	0.25		0.28										
*Moral identity-Symbolization-OCB*	3	1,368	0.29	0.20	0.34	0.21	0.21	0.47	0.08	0.60	9.76***	79.51	88	0.83	0.56
Moral identity-Symbolization-OCBI	1	466	0.39		0.44										
Moral identity-Symbolization-OCBO	1	466	0.25		0.28										
*Moral identification-OCB*	2	788	0.08	0.15	0.11	0.17	−0.09	0.31	−0.08	0.30	4.24 (0.279)	76.39			
Moral identification-OCBI	1	394	0.01		0.01										
Moral identification-OCBO	1	394	0.15		0.21										
*Ethical behavior-OCB*	21	5,729	0.33	0.20	0.39	0.23	0.26	0.52	0.13	0.65	221.87***	90.99	3,120	1.15	0.26
Ethical behavior-OCBI	2	500	0.20	0.11	0.22	0.11	0.13	0.31	0.08	0.36	1.10***	9.20			
Ethical behavior-OCBO	2	500	0.28	0.16	0.32	0.17	0.18	0.46	0.11	0.53	2.55***	60.80			
Ethical behavior-Employee-OCB	6	1,663	0.35	0.20	0.41	0.22	0.30	0.52	0.15	0.67	14.81***	66.24	317	0.23	0.83
Ethical behavior-Employee-OCBI	1	250	0.24		0.26										
Ethical behavior-Employee-OCBO	1	250	0.34		0.37										
Ethical behavior-Customer-OCB	4	1,083	0.24	0.14	0.27	0.15	0.15	0.38	0.09	0.45	8.63***	65.25	61	6.01	0.03
Ethical behavior-Customer-OCBI	1	250	0.15		0.17										
Ethical behavior-Customer-OCBO	1	250	0.21		0.24										
Ethical behavior-Society-OCB	1	333	0.37		0.41										
Ethical behavior-Owner-OCB	1	333	0.36		0.39										
Ethical behavior-Competitor-OCB	1	333	0.37		0.41										
Ethical behavior-Supplier-OCB	1	333	0.34		0.37										
*Ethical behavior intention-OCB*	1	525	0.20		0.25										
*Ethical sensitivity-OCB*	1	98	0.72		0.87										
*Moral courage-OCB*	2	936	0.15	0.10	0.18	0.11	0.10	0.27	0.05	0.31	0.872***	0.00			
*Moral efficacy-OCB*	1	500	0.16		0.20										
*Ethical Orientation of Human Resource Management-OCB*	1	174	0.54		0.61										
*Ethical leadership-OCB*	79	37,130	0.41	0.23	0.49	0.24	0.42	0.57	0.20	0.78	1977.49***	96.06	17,890	3.62	0.00
Ethical leadership-OCBI	20	12,045	0.34	0.15	0.39	0.16	0.30	0.49	0.20	0.58	217.37***	91.26	3,965	1.98	0.06
Ethical leadership-OCBO	21	11,857	0.41	0.22	0.49	0.23	0.38	0.60	0.21	0.77	427.96***	95.33	6,131	3.39	0.00
*Ethical leadership-Promotive-OCB*	1	326	0.41		0.46										
*Ethical leadership-Preventive-OCB*	1	326	−0.14		−0.16										
*Work ethics-OCB*	11	2,818	0.35	0.23	0.45	0.24	0.23	0.67	0.16	0.74	151.56***	93.40	1,028	1.32	0.22
Work ethics-OCBI	1	189	0.16		0.18										
Islamic work ethics-OCB	8	1,605	0.29	0.18	0.34	0.20	0.18	0.50	0.11	0.57	57.11***	87.74	262	0.64	0.55
Islamic work ethics-OCBI	1	189	0.16		0.18										
*Moral reasoning-OCB*	3	348	0.21	0.19	0.26	0.20	0.15	0.37	0.02	0.50	0.90***	0.00			
Moral reasoning-OCBI	3	348	0.21	0.19	0.26	0.20	0.15	0.37	0.02	0.50	0.90***	0.00			
*Ethical climate-OCB*	111	55,903	0.23	0.14	0.29	0.16	0.21	0.36	0.11	0.47	4265.74***	97.30	1,101	2.53	0.01
Ethical climate-OCBI	35	15,745	0.16	0.12	0.20	0.14	0.10	0.30	0.05	0.35	1141.18***	97.00	7,300	2.60	0.01
Ethical climate-OCBO	35	17,987	0.21	0.14	0.28	0.15	0.14	0.42	0.10	0.46	1195.70***	97.41	8,008	0.17	0.86
*Ethical climate-Care oriented-OCB*	11	5,899	0.33	0.19	0.41	0.20	0.27	0.54	0.17	0.65	297.26***	96.60	2,624	3.19	0.01
Ethical climate-Care oriented-OCBI	8	3,563	0.36	0.15	0.48	0.16	0.24	0.72	0.26	0.67	141.78***	95.06	325	2.68	0.04
Ethical climate-Care oriented-OCBO	8	4,218	0.33	0.27	0.40	0.28	0.23	0.57	0.05	0.75	204.08***	96.57	1,090	1.51	0.18
*Ethical climate-Independent judgment oriented-OCB*	17	6,044	0.29	0.17	0.36	0.19	0.17	0.56	0.14	0.58	842.34***	98.10	2,512	1.32	0.21
Ethical climate-Independent judgment oriented-OCBI	8	3,470	0.20	0.22	0.22	0.23	−0.10	0.55	−0.06	0.50	172.28 (0.182)	92.45	324	1.19	0.26
Ethical climate-Independent judgment oriented-OCBI	6	2052	0.51	0.21	0.69	0.25	0.56	0.83	0.42	0.96	256.74*	96.49	418	0.44	0.34
*Ethical climate-Company interest oriented-OCB*	6	1984	0.21	0.15	0.26	0.16	0.07	0.45	−0.59	0.60	45.80***	89.10	121	2.64	0.04
Ethical climate-Company interest oriented-OCBI	3	522	0.12	0.13	0.18	0.15	0.09	0.27	0.01	0.35	2.18***	8.40			
Ethical climate-Company interest oriented-OCBO	1	174	0.16		0.23										
*Ethical climate-Individual interest oriented-OCB*	13	3,570	−0.21	0.19	−0.28	0.20	−0.35	−0.20	−0.54	−0.02	59.60***	79.90	515	1.41	0.19
Ethical climate-Individual interest oriented-OCBI	6	1,288	−0.17	0.17	−0.26	0.20	−0.36	−0.15	−0.48	−0.04	18.64***	73.20	57	5.93	0.02
Ethical climate-Individual interest oriented-OCBO	4	940	−0.22	0.20	−0.28	0.22	−0.48	−0.08	−0.54	−0.02	26.99***	88.90	49	4.00	0.06
*Ethical climate-Regulatory oriented-OCB*	21	9,039	0.28	0.26	0.35	0.28	0.21	0.49	−0.79	0.82	637.54***	96.86	4,088	0.94	0.36
Ethical climate-Regulatory oriented-OCBI	8	4,478	0.33	0.26	0.37	0.29	0.10	0.63	0.04	0.70	560.66**	98.80	178	0.40	0.70
Ethical climate-Regulatory oriented-OCBO	5	2,384	0.44	0.31	0.59	0.33	0.39	0.79	0.19	0.99	128.45***	96.10	81	0.22	0.84
*Ethical climate-Tool oriented-OCB*	9	4,625	−0.15	0.10	−0.19	0.11	−0.25	−0.13	−0.32	−0.06	36.10***	77.80	215	0.62	0.56
Ethical climate-Tool oriented-OCBI	1	250	−0.171		−0.20										
Ethical climate-Tool oriented-OCBO	2	758	−0.17	0.14	−0.22	0.15	−0.33	−0.11	−0.40	−0.04	2.19***	54.40			
*Ethical culture-OCB*	3	972	0.48	0.21	0.57	0.22	0.23	0.90	0.30	0.84	52.75***	96.20	160	0.11	0.93
*Ethical responsibility-Corporate social responsibility-OCB*	30	8,927	0.40	0.19	0.47	0.20	0.39	0.56	0.23	0.71	358.15***	91.90	6,462	0.25	0.81
Ethical responsibility-Corporate social responsibility-OCBI	1	466	0.30		0.33										
Ethical responsibility-Corporate social responsibility-OCBO	1	466	0.28		0.31										
*Ethical responsibility-CSR-society and environment-OCB*	2	399	0.36	0.12	0.41	0.13	0.31	0.51	0.26	0.56	0.29***	0.00			
*Ethical responsibility-CSR-employee-OCB*	2	399	0.36	0.15	0.43	0.17	0.33	0.53	0.24	0.62	0.50***	0.00			
*Ethical responsibility-CSR-customer-OCB*	2	399	0.33	0.15	0.44	0.17	0.24	0.65	0.25	0.63	4.17***	76.00			
*Ethical responsibility-CSR-government-OCB*	1	215	0.48		0.56										
*Ethical values-OCB*	4	1,287	0.45	0.30	0.55	0.33	0.23	0.88	0.17	0.93	97.49***	96.92	242	0.77	0.52
*Moral credits-OCB*	1	674	0.37		0.48										
*Moral credentials-OCB*	1	674	0.56		0.89										
*Moral practice ability-OCB*	15	9,420	0.42	0.22	0.60	0.24	0.40	0.79	0.32	0.88	590.17***	97.63			
Moral practice ability-OCBI	5	3,140	0.47	0.12	0.66	0.15	0.63	0.70	−0.04	0.84	190.22***	97.90			
Moral practice ability-OCBO	5	3,140	0.35	0.20	0.52	0.22	0.20	0.85	0.26	0.78	94.39***	96.82			
*Moral practice ability-Justice-OCB*	3	1884	0.23	0.18	0.29	0.24	0.03	0.56	0.06	0.52	41.45*	95.18			
Moral practice ability-Justice-OCBI	1	628	0.41		0.51										
Moral practice ability-Justice-OCBO	1	628	0.10		0.14										
*Moral practice ability-Prudence-OCB*	3	1884	0.57	0.14	0.76	0.15	0.72	0.79	0.58	0.94	7.04***	71.60			
Moral practice ability-Prudence-OCBI	1	628	0.53		0.76										
Moral practice ability-Prudence-OCBO	1	628	0.47		0.72										
*Moral practice ability-Integrity-OCB*	3	1884	0.69	0.10	0.74	0.12	0.67	0.80	0.61	0.87	19.66***	89.80			
Moral practice ability-Integrity-OCBI	1	628	0.64		0.77										
Moral practice ability-Integrity-OCBO	1	628	0.51		0.67										
*Moral practice ability-Self-control-OCB*	3	1884	0.06	0.10	0.07	0.13	0.02	0.13	−0.06	0.20	3.11**	35.60			
Moral practice ability-Self-control-OCBI	1	628	0.02		0.03										
Moral practice ability-Self-control-OCBO	1	628	0.09		0.12										
*Ethical position-OCB*	3	750	0.27	0.09	0.33	0.10	0.26	0.40	0.21	0.45	0.64***	0.00			
Ethical position-OCBI	1	250	0.29		0.35										
Ethical position-OCBO	1	250	0.23		0.28										
*Moral disengagement-OCB*	6	2,982	−0.30	0.12	−0.34	0.12	−0.45	−0.23	−0.49	−0.19	16.44***	69.58	371	14.22	0.00
Moral disengagement-OCBI	3	1,491	−0.28	0.12	−0.33	0.13	−0.51	−0.14	−0.48	−0.18	10.19***	80.38	90	16.64	0.04
Moral disengagement-OCBO	3	1,491	−0.30	0.13	−0.34	0.14	−0.50	−0.18	−0.51	−0.16	6.24***	67.96	94	12.83	0.05
*Corporate ethics’ perception-OCB*	6	22,926	0.44	0.12	0.53	0.13	0.42	0.64	0.38	0.68	144.10***	96.53			
Corporate ethics’ perception-OCBI	3	11,463	0.42	0.12	0.50	0.14	0.31	0.70	0.35	0.65	111.96***	98.21			
Corporate ethics’ perception-OCBO	3	11,463	0.46	0.15	0.56	0.16	0.43	0.69	0.37	0.75	22.46***	91.09			
*Moral licensing-OCB*	1	297	0.18		0.23										
*Moral deficits-OCB*	1	270	0.29		0.34										
*Moral attentiveness-OCB*	1	270	0.10		0.11										
*Gender-OCB*	95	33,735	0.04	0.18	0.07	0.19	−0.01	0.15	−0.16	0.30	2518.66 (0.069)	97.86	3,663	2.19	0.03
Gender-OCBI	27	9,603	0.08	0.20	0.15	0.22	−0.06	0.36	−0.11	0.41	837.34 (0.174)	96.90	594	1.10	0.28
Gender-OCBO	19	7,111	0.13	0.20	0.22	0.23	0.00	0.45	−0.04	0.48	1246.44*		1843	0.88	0.39
*Age-OCB*	95	33,735	0.04	0.17	0.07	0.18	−0.01	0.15	−0.15	0.29	1704.79 (0.069)	94.90	6,087	2.11	0.04
Age-OCBI	22	7,164	0.14	0.11	0.19	0.13	0.03	0.35	0.05	0.33	1366.01**	98.50	909	1.16	0.26
Age-OCBO	17	6,178	0.12	0.10	0.17	0.14	0.01	0.34	0.04	0.30	802.17*	97.88	1,288	1.27	0.22
*Education-OCB*	67	22,539	0.02	0.15	0.02	0.16	0.00	0.04	−0.17	0.21	111.08 (0.089)	48.69	58	0.49	0.62
Education-OCBI	11	3,259	−0.01	0.22	0.06	0.24	0.01	0.11	−0.22	0.34	12.98*	22.93	11	2.08	0.07
Education-OCBO	12	3,474	0.07	0.25	0.08	0.25	0.03	0.12	−0.24	0.40	13.88***	20.77	30	1.60	0.14
*Tenure-OCB*	67	24,355	0.00	0.27	−0.02	0.28	−0.11	0.06	−0.37	0.33	1854.65 (0.571)	96.39	476	1.97	0.05
Tenure-OCBI	19	6,983	−0.02	0.23	−0.04	0.24	−0.17	0.10	−0.33	0.25	381.31 (0.601)	95.28	56	1.49	0.15
Tenure-OCBO	10	4,276	−0.18	0.41	−0.34	0.45	−0.79	0.11	−0.87	0.19	800.95 (0.136)	98.75	1,158	1.26	0.24

*p < 0.05, **p < 0.01, ***p < 0.005.

### 4.1 Results of meta-analysis

As detailed in Table 1, moral identity (OCB *ρ* = 0.51, OCBI *ρ* = 0.51, OCBO *ρ* = 0.54), moral identification (OCB *ρ* = 0.11, OCBI *ρ* = 0.01, OCBO *ρ* = 0.21), ethical behavior (OCB *ρ* = 0.39. OCBI *ρ* = 0.22, OCBO *ρ* = 0.32), ethical behavior intention (*ρ* = 0.25), ethical sensitivity (*ρ* = 0.87), moral courage (*ρ* = 0.18), moral efficacy (*ρ* = 0.20), ethical orientation of Human Resource Management (*ρ* = 0.61), ethical leadership (OCB *ρ* = 0.49, OCBI *ρ* = 0.39, OCBO *ρ* = 0.49), work ethics (*ρ* = 0.45), moral reasoning (*ρ* = 0.26), ethical climate (OCB *ρ* = 0.29, OCBI *ρ* = 0.20, OCBO *ρ* = 0.28), ethical culture (*ρ* = 0.57), ethical responsibility (OCB *ρ* = 0.47, OCBI *ρ* = 0.33, OCBO *ρ* = 0.31), ethical values (*ρ* = 0.55), moral credits (*ρ* = 0.48), moral credentials (*ρ* = 0.89), moral practice ability (OCB *ρ* = 0.60, OCBI *ρ* = 0.66. OCBO *ρ* = 0.52), ethical position (OCB *ρ* = 0.33, OCBI *ρ* = 0.35, OCBO *ρ* = 0.28), corporate ethics’ perception (OCB *ρ* = 0.53, OCBI *ρ* = 0.50, OCBO *ρ* = 0.56), moral licensing (*ρ* = 0.23), moral deficits (*ρ* = 0.34), moral attentiveness (*ρ* = 0.11), and education (OCB *ρ* = 0.02, OCBI *ρ* = 0.06, OCBO *ρ* = 0.08) were significantly positively associated with OCB (95% CI excluding zero). Conversely, moral disengagement (ρ = −0.34) was significantly negatively associated with OCB (Detailed dimensional results for OCB-I and OCB-O are provided in Table 1).

According to Cohen’s guidelines for organizational research, a ρ of 0.10 represents a small effect, 0.30 a medium effect, and 0.50 a large effect. The variation in effect size among the ethical factors is substantial and theoretically meaningful. Highly internalized personal traits (e.g., moral identity, *ρ* = 0.51) and macro-level organizational environments (e.g., ethical cultures, *ρ* = 0.57) have a very strong relationship with OCB. In contrast, more distant or generalized constructs like a general ethical climate (*ρ* = 0.29) show moderate effects - that is, generalized climates need to be translated into proximal leadership or culture in order to matter. Notably, the extremely high correlation of moral credentials (*ρ* = 0.89) approaches one, indicating that there is a risk of tautology between the main studies’ operationalization of credentialing and citizenship. To maintain theoretical discipline, findings for constructs with a small number of underlying studies (e.g., *k* < 5) must be interpreted with caution, as their magnitudes may partly reflect tautological measurement in the primary studies rather than substantive distinctiveness, it is requiring cautious interpretation.

Notably, dimensional subtleties appeared in some constructs. For example, although overall ethical leadership has a strong positive effect on OCB, when you break that down to preventative ethical leadership there is a slight negative relationship with OCB. Likewise, although the overall ethical climate was positively correlated with OCB, the individual-interest-oriented ethical climate-individual interest oriented had a significant negative correlation with OCB (OCB *ρ* = −0.28, OCBI *ρ* = −0.26, OCBO *ρ* = −0.28), and the instrumental/tool -oriented ethical climate also had a significant negative correlation with OCB (OCB *ρ* = −0.19, OCBI *ρ* = −0.20, OCBO *ρ* = −0.22). Moral identification had no obvious connection with gender, age, length of service and OCB (95% CI including 0, as seen in Table 1).

Crucially, the systematic breakdown of heterogeneity metrics (I^2^, Q statistics) justified our methodology. In most of our main effects, significant Q statistics and I2 values above 75 percent can be seen (details in Table 1). This important heterogeneity indicates that the difference in effect sizes of the main studies is actually due to true differences in theory, situation, and method, rather than just random sampling error. Because these effect sizes are highly variable, deploying a random-effects model was theoretically and mathematically requisite; it allows us to generalize our pooled effect sizes across a distribution of true effects rather than assuming a single, universal population parameter. This high heterogeneity also mandated the rigorous moderator analyses detailed below. Therefore, H1a, H1b, H1c, H1d, H1e, H1f, H1g, H1h, H2a, H2b, H2c, H2d, H2e, H2f, H2g, H3a, H4a, H4b, H4c, H5a, H5b, H5c, H5d, and H6c were supported.

### 4.2 Results of moderator analysis

We note that the majority of Failsafe N (Rosenthal) values are greater than the 5 K + 10 threshold (where *K* is the number of included studies). Furthermore, intercepts for Egger regression are mostly equal to or close to zero, and the majority of *p*-values are not significant. In ethical responsibility and work ethics, Failsafe N were 1,028 and 6,462, respectively, (much greater than their respective 5 K + 10, thresholds of 65 and 160), with non-significant Egger’s *p*-values of 0.81 and 0.22. Therefore, we can conclude that, publication bias, even if it exists, is negligible and can be disregarded.

In order to explore potential boundary conditions explaining the aforementioned heterogeneity, we selected variable relationships exhibiting high heterogeneity (*I*^2^ greater than 75%) and that also met at least two contextual conditions (i.e., varying across different studies, sample populations, cultural backgrounds, gender ratios, or funding information) to conduct moderating effect analyses. Table 2 shows the moderators’ results. Rather than being peripheral details, these moderator findings reveal critical boundary conditions. First, publication year positively moderated specific relationships (e.g., ethical behavior–OCB, point estimate = 0.04; ethical leadership–OCB, point estimate = 0.01, *p* < 0.001). This indicates that there is a time evolution of the work place: In the past two decades since corporate global governance tightening, the drive of ethical leadership on citizenship norms has increased. Second, the cultural background (national development level) demonstrated a significant regulation effect with point estimate between 0.30 and 0.50. This suggests that the macro-economic and institutional context of a country plays a significant role. In particular, there is a stronger relationship among ethical elements and OCB in developing countries, a possible reason might be that in these situations, where organizations tend to have greater uncertainty about the future, ethics-led leadership and climates may become more important signs of organization’s reliability and authenticity; Therefore, they can have a stronger impact on employees’ behavior.

**Table 2:** The moderating effect of ethical factors and OCB.

Moderators	Relationships	Models	*𝑘*	Point estimate	95% CI		Q	Qwithin	Qbetween	*p* value
Publication years	Moral identity-OCB	Regression	24	0.04	0.00	0.08	3.43			0.060
*Gender ratio (male %)*		Fixed	24	0.36	0.34	0.38	331.11	304.10	27.00	0.000
<49%		Fixed	5	0.35	0.31	0.40	50.08			0.000
49%–51%		Fixed	11	0.40	0.37	0.43	200.40			0.000
>51%		Fixed	8	0.30	0.26	0.34	53.62			0.000
*National development level*		Fixed	24	0.36	0.34	0.38	331.11	1.35	331.11	0.000
Developed		Fixed	10	0.38	0.34	0.41	98.85			0.000
Developing		Fixed	14	0.35	0.33	0.38	230.91			0.000
*Sample population*		Fixed	24	0.36	0.34	0.38	331.11	293.09	38.02	0.000
Employees		Fixed	15	0.40	0.37	0.43	180.34			0.000
Managers		Fixed	2	0.39	0.28	0.49	0.04			0.000
Mixed		Fixed	6	0.34	0.31	0.37	112.71			0.000
Students		Fixed	1	0.14	0.05	0.23	0.00			0.002
*Funding*		Fixed	24	0.36	0.34	0.38	331.11	329.71	1.40	0.000
No		Fixed	14	0.35	0.32	0.38	156.83			0.000
Yes		Fixed	10	0.37	0.35	0.40	172.87			0.000
Publication years	Ethical behavior-OCB	Regression	21	0.04	0.02	0.06	10.47			0.001
*Gender ratio (male %)*		Fixed	21	0.30	0.28	0.33	221.87	186.22	35.65	0.000
<49%		Fixed	4	0.45	0.39	0.50	93.76			0.000
49%–51%		Fixed	6	0.33	0.29	0.37	3.92			0.000
>51%		Fixed	11	0.25	0.22	0.28	88.54			0.000
*National development level*		Fixed	21	0.30	0.28	0.33	221.87	189.59	32.27	0.000
Developed		Fixed	4	0.44	0.39	0.49	86.45			0.000
Developing		Fixed	17	0.27	0.24	0.30	103.14			0.000
*Sample population*		Fixed	21	0.30	0.28	0.33	221.87	220.54	1.33	0.000
Employees		Fixed	19	0.30	0.27	0.32	216.15			0.000
Managers		Fixed	2	0.34	0.27	0.41	4.38			0.000
Mixed		Fixed	-	-	-	-	-	-	-	-
Students		Fixed	-	-	-	-	-	-	-	-
*Funding*		Fixed	21	0.30	0.28	0.33	221.87	218.14	3.73	0.000
No		Fixed	14	0.29	0.26	0.32	80.03			0.000
Yes		Fixed	7	0.34	0.30	0.38	138.11			0.000
Publication years	Ethical leadership-OCB	Regression	79	0.01	−0.014	0.02			0.19	0.000
*Gender ratio (male %)*		Fixed	79	0.33	0.32	0.34	1977.49	1813.63	163.86	0.000
<49%		Fixed	17	0.37	0.35	0.40	274.97			0.000
49%–51%		Fixed	20	0.42	0.40	0.43	366.99			0.000
>51%		Fixed	42	0.28	0.27	0.30	1171.67			0.000
*National development level*		Fixed	79	0.33	0.32	0.34	1977.49	1489.02	488.47	0.000
Developed		Fixed	7	0.19	0.17	0.21	15.16			0.000
Developing		Fixed	72	0.41	0.40	0.42	1473.86			0.000
*Sample population*		Fixed	79	0.33	0.32	0.34	1977.49	1498.89	478.61	0.000
Employees		Fixed	69	0.41	0.40	0.42	1153.99			0.000
Managers		Fixed	3	0.47	0.41	0.53	94.22			0.000
Mixed		Fixed	8	0.21	0.19	0.22	250.68			0.000
Students		Fixed	-	-	-	-	-	-	-	-
*Funding*		Fixed	79	0.33	0.32	0.34	1977.49	1974.01	3.48	0.000
No		Fixed	62	0.33	0.32	0.34	1778.23			0.000
Yes		Fixed	17	0.31	0.29	0.33	195.78			0.000
Publication years	Work ethics-OCB	Regression	11	−0.02	−0.06	0.03			0.53	0.464
*Gender ratio (male %)*		Fixed	11	0.38	0.34	0.41	151.56	141.49	10.07	0.000
<49%		Fixed	3	0.32	0.25	0.39	7.87			0.000
49%–51%		Fixed	4	0.34	0.28	0.39	26.38			0.000
>51%		Fixed	4	0.43	0.39	0.48	107.23			0.000
*National development level*		Fixed	11	0.38	0.34	0.41	151.56	151.56	0.00	0.000
Developed		Fixed	11	0.38	0.34	0.41	151.56			0.000
Developing		Fixed	-	-	-	-	-	-	-	-
*Sample population*		Fixed	11	0.38	0.34	0.41	151.56	117.66	33.89	0.000
Employees		Fixed	10	0.40	0.37	0.43	117.66			0.000
Managers		Fixed	1	0.00	−0.14	0.14	0.00			1.000
Mixed		Fixed	—	—	—	—	—	—	—	—
Students		Fixed	—	—	—	—	—	—	—	—
*Funding*		Fixed	11	0.38	0.34	0.41	151.56	141.69	9.86	0.000
No		Fixed	8	0.35	0.31	0.38	62.19			0.000
Yes		Fixed	3	0.46	0.40	0.51	79.50			0.000
Publication years	Ethical climate-OCB	Regression	111	0.03	0.02	0.04	45.19			0.000
*Gender ratio (male %)*		Fixed	111	0.31	0.30	0.32	4089.99	3844.42	245.57	0.000
<49%		Fixed	41	0.22	0.20	0.24	1021.02			0.000
49%–51%		Fixed	24	0.40	0.38	0.41	1598.43			0.000
>51%		Fixed	46	0.30	0.29	0.31	1224.97			0.000
*National development level*		Fixed	111	0.31	0.30	0.32	4089.99	3676.30	413.68	0.000
Developed		Fixed	18	0.42	0.41	0.43	278.54			0.000
Developing		Fixed	93	0.26	0.25	0.27	3,397,77			0.000
*Sample population*		Fixed	111	0.31	0.30	0.32	4089.99	3752.47	337.51	0.000
Employees		Fixed	96	0.27	0.26	0.28	3238.57			0.000
Managers		Fixed	5	0.25	0.16	0.34	0.00			0.000
Mixed		Fixed	2	0.40	0.38	0.41	21.50			0.000
Students		Fixed	8	0.46	0.43	0.48	392.87			0.000
*Funding*		Fixed	111	0.31	0.30	0.32	4089.99	3583.14	506.85	0.000
No		Fixed	82	0.36	0.35	0.37	2824.27			0.000
Yes		Fixed	29	0.15	0.13	0.17	758.87			0.000
Publication years	Ethical responsibility-OCB	Regression	30	0.02	0.00	0.04	5.14			0.023
*Gender ratio (male %)*		Fixed	30	0.39	0.37	0.40	358.15	332.25	25.90	0.000
<49%		Fixed	8	0.42	0.38	0.45	142.60			0.000
49%–51%		Fixed	4	0.37	0.32	0.41	139.31			0.000
>51%		Fixed	18	0.38	0.36	0.41	50.34			0.000
*National development level*		Fixed	30	0.39	0.37	0.40	358.15	355.60	2.57	0.000
Developed		Fixed	3	0.43	0.37	0.49	33.65			0.000
Developing		Fixed	27	0.38	0.36	0.40	321.95			0.000
*Sample population*		Fixed	30	0.39	0.37	0.40	358.15	308.83	49.32	0.000
Employees		Fixed	26	0.40	0.38	0.42	308.61			0.000
Managers		Fixed	1	0.59	0.52	0.65	0.00			0.000
Mixed		Fixed	3	0.28	0.23	0.32	0.22			0.000
Students		Fixed	—	—	—	—	—	—	—	—
*Funding*		Fixed	30	0.39	0.37	0.40	358.15	309.88	48.27	0.000
No		Fixed	23	0.35	0.33	0.37	135.78			0.000
Yes		Fixed	7	0.49	0.45	0.52	174.10			0.000

*p < 0.05, **p < 0.01, ***p < 0.005.

### 4.3 Results of impact mechanism

**Figure 3:**
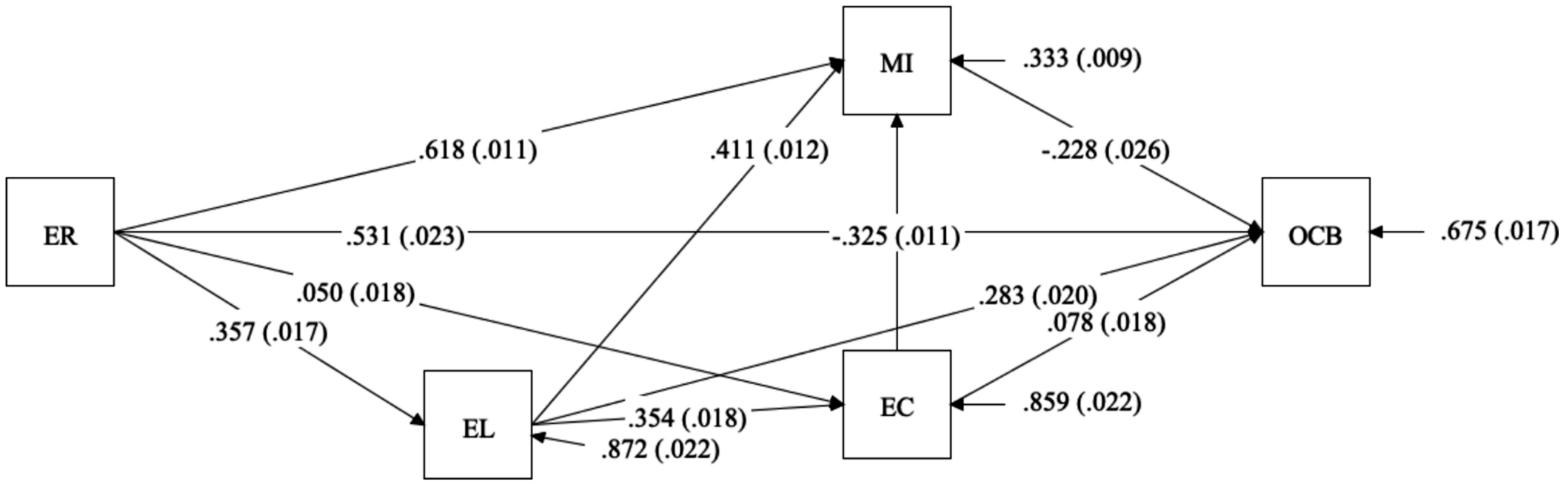
Paths of multiple chain mediation models for OCB. EC, Ethical climate; EL, Ethical leadership; ER, Ethical responsibility; MI, Moral identity; OCB, Organization citizenship behavior. Harmonic mean = 3,047, Chi-Square = 5422.198, CFI = 1.000, TLI = 1.000, SRMR = 0.000, *p* < 0.001.

In terms of the dual and multi-chain pathways linking ethical responsibility to OCB (Figure 3). Table 3 presents us the correlation matrix for the 5 study variables used in the MASEM analysis. Our hypothesized model demonstrated excellent fit to the data (Harmonic mean = 3,047; *χ*^2^ = 5422.198; CFI = 1.000; TLI = 1.000; SRMR = 0.000). These good fit indices (Figure 3) indicate that our proposed dual-pathway structure adequately reproduces the underlying correlation matrix. It is important to note that in MASEM, where the input is a synthesized correlation matrix, such perfect fit often indicates that the proposed model is exactly identified or fully accounts for the target correlations, confirming that the hypothesized relationships are structurally consistent with the aggregate data. Indeed, our specified theoretical model (Figure 3) includes all possible recursive paths among the five variables, rendering it a fully saturated (just-identified) model. Consequently, the perfect fit indices (CFI = 1.000; TLI = 1.000; SRMR = 0.000) are a mathematical artifact of this saturation rather than an independent validation of empirical fit. To validate our specific indirect-effect estimates under conditions of strong heterogeneity, we compared our saturated model against a nested alternative model (a full mediation model constraining the direct relationship between ethical responsibility and OCB to zero). The alternative constrained model exhibited significantly poorer fit (∆*χ*^2^ test significant at *p* < 0.01), supporting the retention of our dual-pathway specification. Furthermore, the indirect effects were estimated using the conservative harmonic mean sample size to ensure that massive-sample studies did not disproportionately skew the mediational pathways.

**Table 3:** Meta-analytic correlation matrix for OCB.

Variables	OCB	EC	MI	EL	ER
OCB	1				
EC	0.287*** (116, 57,185)	1			
MI	0.292*** (24, 7,588)	−0.064*** (7, 2,578)	1		
EL	0.386*** (79, 37,130)	0.510*** (10, 8,584)	0.372*** (7, 4,518)	1	
ER	0.485*** (30, 8,927)	0.707*** (2, 602)	0.176*** (9, 2,390)	0.357*** (16, 5,321)	1

Harmonic mean = 3,047; the data in parentheses are k, *N*, ***p < 0.005.

As shown in Table 3, ethical climate and ethical leadership positively mediated the link between ethical responsibility and OCB, for example, the estimated path for CSR → EC → OCB is 0.004 (*p* = 0.020), and the estimated path for CSR → EL → OCB is 0.101 (*p* < 0.001), the estimated indirect path value for CSR → EC → MI → OCB is 0.004 (*p* = 0.020), the estimated path value for CSR → EL → EC → OCB is 0.010 (*p* < 0.001), and the estimated path value for CSR → EL → EC → MI → OCB is 0.009 (*p* < 0.001). When compared with each other, it is evident that the adverse indirect effect of CSR on organizational performance via moral identity (−0.141) is relatively greater in scale than its positive counterpart mediated by ethical leadership (0.101). This indicates that the psychological alienation caused by improperly internalized CSR can effectively neutralize or even overpower the structural benefits of ethical leadership.

Conversely, moral identity negatively mediated the link between ethical responsibility and OCB, similar to its role between ethical leadership and OCB, for example, the estimated path value for CSR → MI → OCB is −0.141 (*p* < 0.001), and the estimated path value of CSR → EL → MI → OCB is −0.033 (*p* < 0.001), and the indirect effects in Table 3 show that the significance level of the product terms of indirect effects of these paths is zero, that is, the seven indirect effects are all statistically significant. Therefore, H3b and H3c were supported.

Table 4 presents the path coefficients for the multiple-chain mediated effects estimated by the Mplus software. Based on the coefficients in Figure 3, we calculated the specific indirect effects as follows: the CSR → MI → OCB is 0.618 × (−0.228) = −0.141; the CSR → EC → OCB is 0.050 × 0.078 = 0.004; and the CSR → EL → OCB is 0.357 × 0.283 = 0.101; the estimated indirect effect of CSR → EC → MI → OCB is 0.050 × (−0.325) × (−0.228) = 0.004; the CSR → EL → MI → OCB is 0.357 × 0.410 × (−0.228) = −0.033; the CSR → EL → EC → OCB is 0.357 × 0.354 × 0.078 = 0.010, and the CSR → EL → EC → MI → OCB is 0.357 × 0.354 × (−0.325) × (−0.228) = 0.009. Consistent with the indirect effect estimation results in Table 3, all calculated indirect effects were statistically significant. Therefore, the impact of ethical responsibility on OCB via these dual pathways was verified.

**Table 4:** Path coefficients of multiple chain mediation models for OCB.

Paths	Estimate	SE	*Z*	*p* value
Total	0.485	0.02	30.61	0.000
Total indirect	−0.046	0.02	−2.60	0.009
Direct ER→OCB	0.531	0.02	25.55	0.000
ER→MI→OCB	−0.141	0.02	−8.73	0.000
ER→EC→OCB	0.004	0.00	2.32	0.020
ER→EL→OCB	0.101	0.01	11.78	0.000
ER→EC→MI→OCB	0.004	0.00	2.62	0.009
ER→EL→MI→OCB	−0.033	0.00	−7.94	0.000
ER→EL→EC→OCB	0.010	0.00	4.15	0.000
ER→EL→EC→MI→OCB	0.009	0.00	7.29	0.000

### 4.4 Results of robustness check

Our robustness analysis was tested using three methods: one study removed, cumulative analysis, and funnel plot. The test results are shown in Supplementary materials, and the results show that our meta-analysis results are reliable, robust, and unchanged. In addition, we supplemented the robustness of each subgroup variable with Fail safe *N* (Rosenthal) and Egger’s test, and the results detailed Table 1. The results confirm that each subgroup analysis is also robust and reliable.

## 5 Discussion

To the best of our knowledge, we have conducted the first systematic meta-analysis to better understand how ethics impact OCB at this scale and complexity. We classified and integrated identical or conceptually similar constructs (e.g., different types of ethical factors or organizational citizenship behaviors) to synthesize prior empirical work (*k* = 125, *N* = 158,336). This allowed us to reveal the complex mechanisms underlying the relationships between different types of ethical factors and OCB and to robustly estimate their linkages.

First, the results demonstrate that moral identity, ethical behavior, ethical behavior intention, ethical sensitivity, moral courage, moral efficacy, ethical orientation of Human Resource Management, ethical leadership, work ethics, moral reasoning, ethical climate, ethical culture, ethical responsibility, ethical values, moral credits, moral credentials, moral practice ability, ethical position, corporate ethics’ perception, moral licensing, moral deficits, moral attentiveness, and education are significantly and positively related to OCB. Our findings provide a more comprehensive empirical baseline compared to previous meta-analyses that were often narrower in scope. For instance, Ogunfowora et al. (2022) conducted a valuable meta-analysis focusing specifically on moral disengagement. Our findings corroborated their conclusion that moral disengagement significantly negatively predicts OCB (*ρ* = −0.34). However, we have fully explored its mechanism of action as a major behavioral inhibitor instead of merely focusing on one or two similar variables our study takes us far beyond that single construct through a concurrent investigation into another 27 ethics factors. We linked the context of moral disengagement to those positive factors, such as moral identity (*ρ* = 0.51) and ethical culture (*ρ* = 0.57). Our synthesis provides a comprehensive nomological network. This allows researchers to gauge the relative impact of an ethical inhibitor against an ethical driver of OCB.

In addition, this paper further extends that of Wang and Wang (2018), which mainly examined the linkages among ethical leadership, organizational commitment or others under specific circumstances; it focused on a particular field such as health care. Although it is possible to explore a broad understanding of the ethical dilemma in various industries theoretically. Incorporating 125 studies involving over 158,000 people from various ethical perspectives to strengthen the generalization of our baseline among diverse industries. More importantly, whereas many previous meta-analyses focused on single or bivariate association(s), our MASEM is a major improvement in methodology, we can draw the complicated, co-occurring dual pathways (such as: ethical climate promotes responsibility, but moral identity inhibits it) of how ethical responsibility influences OCB through structure, which were not able to be seen by previous meta-analysis.

As for our non-significant main effects, gender showed a complicated pattern. Although according to social role theory there are likely behavioral differences; currently the effect is relatively weak overall. Typically, gender differences are manifested in the form of citizens’ behavior rather than their overall quantity; female citizens prefer inter-personal OCBI alignment with community role model (Kaur and Kang, 2023; Wang et al., 2025), while male citizens have a preference for OCB-O or civic virtue. Therefore, because the primary studies have various dimensions of operationalizing OCB and they are varied, this may offset each other, leading to no significant meta-analysis result.

Similarly, the non-significant results of age and organizational tenure are also related to various complex motivations at different career levels, mostly, early-career or low tenured workers are instrumental in engaging in citizen behavior to build good relationships with others, and to gain a favorable reputation (Xu et al., 2022). Conversely, older or long-tenured staff often display OCB out of a sense of deep affection and duty (Wang et al., 2022). Moreover, with the increase in age, some people may have a certain degree of uncertainty regarding how their performance should be evaluated; at this point, fulfilling these obligations has become an obligation imposed upon them. Due to these changes in fundamental motivations, despite consistent behavioral output, the linear relationship between time-based measures and OCB within a heterogeneous aggregate sample is obscured.

Finally, the non-significant main effect of moral identification suggests that it differs from internalized constructs like moral identity conceptually and empirically. According to social identity theory, moral identification is an interpersonal relationship and generally needs a certain, important target, such as an ethical role model or a firm corporate ethical atmosphere, to be triggered in line with common moral norms (Qaiser and Zhang, 2023). Without a clear moral “catalyst” or boundary condition for this identification to anchor upon, it is attenuated and less likely to directly translate into discretionary behavioral outcomes (Xu et al., 2022). Thus, our findings suggest that moral identification should be conceptualized primarily as a contingent variable that relies on contextual triggers, rather than a universal, direct predictor of OCB.

### 5.1 Theoretical implications

The theoretical implications of this study are as follows: First, our findings systematically validate and extend SLT, ethical behavior can be learned through observing and modeling it within organizations. The association between ethics and OCB can be better elucidated by examining the impact of ethical leadership and modeling ethical behaviors. In SLT, ethical leadership can serve as a powerful predictor of employees’ ethical behavior within an organization (Bandura, 1977; Mansur et al., 2020; Ruiz-Palomino and Martínez-Baños, 2014; Shahzad et al., 2025; Shin, 2012). In organizational contexts, individuals acquire behaviors by observing and imitating credible role models. Our meta-analytic results confirm that highly visible ethical modeling specifically ethical leadership (𝜌 = 0.49) and ethical climate (𝜌 = 0.29) serves as a primary structural driver of discretionary behavior. Furthermore, our MASEM results visually map this observational learning mechanism, demonstrating that CSR shows a positive indirect relationship with OCB specifically when mediated by ethical leadership and ethical climate (e.g., CSR → EL → OCB path = 0.101, *p* < 0.001). When employees observe ethical leadership behavior and operate within an ethical climate, they are more likely to emulate this normative behavior and consequently engage in OCB. Bandura (1986) argued that modeling has a greater impact on behavior when it is perceived as having a positive outcome. Thus, our results confirm that when employees observe that ethical behavior is embedded and modeled within an organization, they are significantly more likely to emulate it and engage in prosocial OCB. We advance SLT by demonstrating that modeling is not a passive transfer of values; rather, macro-level ethics (CSR) remain behaviorally inert unless actively translated and demonstrated by proximal role models (i.e., leaders).

Second, our integration of 28 distinct ethical factors provides a highly nuanced empirical extension of Social Exchange Theory, reciprocity in social interactions drives human behavior (Blau, 1964; Cropanzano and Mitchell, 2005). By contrasting ethical promoters with ethical inhibitors, our findings illustrate the dual edges of social exchange. On the positive side, our results show that strong ethical cultures (*ρ* = 0.57) and work ethics (*ρ* = 0.45) engender high-quality reciprocal exchange (Mayer et al., 2012; Shahzad et al., 2025).

However, our study generates critical new theoretical insights by using SET to explain our most paradoxical findings: the negative association of individual-interest (*ρ* = −0.28) and tool-oriented ethical climates (*ρ* = −0.19) with OCB. Traditionally, researchers assume “ethical climates” are universally positive. These particular sub-climates fundamentally do not conform to the norms of SET. If the organizations’ culture exhibits instrumentality, self-interest and rigid rules of operation to avoid consequences in zero-sum environments. This severs the socio-emotional, reciprocal psychological contract necessary for discretionary effort. Consequently, employees are more likely to withhold OCB to protect their own resources. Similarly, moral disengagement exhibited a strong inhibitory relationship with OCB (*ρ* = −0.34), acting as a cognitive mechanism that justifies the withholding of reciprocal exchange (Ogunfowora et al., 2022; Trevino and Weaver, 2001; Yam et al., 2017).

Third, our MASEM results revealing the complex, multi-pathway effects of CSR enrich SIT: social identity of individuals within an organization significantly influences their attitudes and behaviors (Ellemers et al., 2004). Wang and Wang (2018) previous research implied that there was generally a positive correlation between ethics and identity, our results revealed a more nuanced reality. When CSR ethically climate is built successfully, it can effectively promote OCB well through the moral values of employees.

Crucially, we found an equally important inhibitory pathway: CSR has a negative effect on OCB via moral identity (CSR → MI → OCB path = −0.141, *p* < 0.001). How might an ethical obligation actively inhibit citizenship behavior? From a view of SIT, this is explained by the psychological mechanism of perceived corporate hypocrisy. When organizations get involved with large -scale CSR projects disconnected from their daily climate and leadership activities, employees with strong moral identities perceive these initiatives as hypocritical green-washing. This experience fails to foster identity congruence and actually alienates employees who have a high level of moral identity. It creates a moral boundary-drawing effect wherein the employee psychologically disassociates themselves from the organization in order to preserve their moral self-concept. Having uncovered this constraint, we have thereby advanced the foundation of SIT by showing that organizational moral identity requires genuine structurally supported ethical integration rather than mere policy signaling. When the theoretical integration of SLT, SET and SIT is viewed holistically coherent framework emerges: leaders must demonstrate genuine and mutually supportive ethical behaviors; otherwise, employees might become cynical and withdraw their discretionary behaviors (Aquino and Reed, 2002; Shahzad et al., 2025).

### 5.2 Practical implications

From a pragmatic perspective, by leveraging our comprehensive meta-analytic dataset encompassing 28 ethical factors, it is possible to eschew generalized recommendations and instead generate specific, prioritized recommendations for managers. Specifically, these guidelines aim to identify which ethical interventions are likely to yield the highest return on investment (ROI). Given that OCB constitutes discretionary, extra-role activities capable of enhancing operational effectiveness (Meyer et al., 2002; Shahzad et al., 2025), carefully treating the integrity-OCB-outcomes chain as a plausible pathway for organizational success emphasizes that ethics should be addressed at two levels: as a regulatory obligation and as a strategic resource for both private and public policy (Saputra et al., 2026).

First, based on the relative magnitude of our effect sizes, organizations should focus more on macro-systemic organizational structures than on individual trait screening. The interventions that aim at cultivating a comprehensive ethical culture and developing the moral practice capability of employees achieve the best ROIs for generating OCB, respectively (*ρ* = 0.57; *ρ* = 0.60). On the other hand, screening candidates for a general moral identity trait in isolation (*ρ* = 0.11) yields almost no behavioral return. Therefore, Human Resource (HR) departments should pivot their budgets from generic compliance testing toward immersive, scenario-based training that enhances active moral practice and ethical leadership (*ρ* = 0.49) (Meyer et al., 2002; Podsakoff et al., 2000; Shahzad et al., 2025). Hence, senior executives should abandon the notion that ethical management is of secondary importance, fully recognize that ethical practices can also generate tangible operational benefits, and consequently define them as a critical strategic resource. Managing increased OCB requires a company to have a clear understanding of its intended outcomes and desirable actions. For a manager, fostering OCB is functionally equivalent to managing organizational efficiency. To optimize work processes and results, managers must identify which civic behaviors (e.g., OCB-I vs. OCB-O) are most beneficial and contribute to achieving core organizational goals.

Second, practitioners must anticipate and navigate significant implementation challenges, primarily the misalignment between ethical directives and organizational incentive structures. Our findings demonstrate that instrumental, “tool-oriented” ethical climates actively suppress OCB (*ρ* = −0.19). In highly competitive, sales-driven industries, or decentralized multinational corporations, attempts to foster an ethical culture will be actively undermined if Key Performance Indicators (KPIs) strictly reward bottom-line financial results at all costs. To overcome this implementation hurdle, organizations must fundamentally overhaul performance management systems to explicitly evaluate and reward ethical conduct and prosocial mentoring, rather than merely punishing compliance failures.

Third, our moderator analyses indicate that these recommendations must be tailored across different cultural and organizational contexts. In highly developed economies, ethical interventions might need to emphasize individual-level ethical leadership and personal moral reasoning to resonate with a context that values individual agency. Conversely, in developing economies, where our moderators revealed amplified effects, interventions should prioritize the establishment of group-level ethical climates and collective work ethics, as the normative organizational environment likely exerts a much stronger influence on discretionary behavior in these contexts.

Finally, as demonstrated by our MASEM results, macro-level CSR only translates into positive employee behavior when mediated by authentic ethical leadership and an ethical climate. Therefore, C-suite executives must actively model ethical behavior to set the “tone at the top,” ensuring that CSR initiatives are integrated into daily internal operations. If an organization promotes external philanthropic CSR while simultaneously harboring toxic, unsupportive internal leadership, it risks triggering the “hypocrisy penalty” identified in our dual-pathway model. This perceived “greenwashing” alienates employees with strong moral identities (*ρ* = −0.141) and drastically reduces the exact citizenship behaviors the organization wishes to promote.

### 5.3 Limitations and future directions

The study must be acknowledged that there are some limitations. First, our meta-analysis results are based on an estimation of the correlation between variables extracted from cross-sectional primary studies; therefore, it is not possible to establish strict causal relationships between ethics and OCB. Moreover, although ethics and morality possess nuanced conceptual distinctions in philosophy, we did not empirically distinguish between them empirically in this synthesis, which limits the theoretical scope of our research. Longitudinal or experimental design studies should be conducted in the future to explain the more detailed impact mechanisms of ethics on OCB from the perspective of strict causal inference and to explore the differential impacts of strictly defined moral versus ethical constructs.

Second, the number of original studies (*k*) for some relationships is relatively small, such as for moral identification, ethical behavior intention, moral courage, and ethical culture, etc. It is worth noting that the sample sizes for these specific factors remain limited (e.g., *k* < 10), and therefore, the results derived from these smaller sample studies are more susceptible to second-order sampling errors. Future researchers and practitioners should interpret these specific effect sizes with caution. We recommend that these findings be continuously updated via future meta-analyses as more empirical data becomes available to render the estimates more robust and accurate.

Third, the included studies were mostly cross-sectional in design and used similar structured instruments for the measurement of variables, relying heavily on self-report surveys. These studies cannot escape the effects of endogenous biases, generalizations error and perception bias (such as social desirability tendency), etc. In response to the above problems in this study, future research may need to adopt an intervention-oriented or longitudinal Design approach, include multiple rating sources (supervisor evaluation of OCB), etc.

Finally, further research will need to delve into the specific paths and mechanisms underlying the results presented in this paper; by adjusting other boundary variables and contexts, it is hoped that a more complete picture can be obtained about how, why and when ethics influences OCB. Faced with this situation, in the future research efforts can be devoted to exploring whether or not these ethics to OCB relationships operates under different institutional contexts outside the public sector (Syamsir et al., 2026).

## 6 Conclusion

Ethical constructs have been identified as a direct, effective, and economical way to influence organizational citizenship behavior (OCB). Although numerous studies have demonstrated the critical role of ethical factors in inhibiting or promoting OCB, a comprehensive quantitative synthesis remains lacking. Therefore, we conducted a meta-analysis to quantitatively synthesize 125 independent studies (28 ethical factors, 331 effect values, and 158,336 participants) and propose a theoretical framework for the relationship between ethics and OCB. The results revealed that 24 ethics factors were significantly and positively related to OCB, whereas moral disengagement was significantly and negatively associated with OCB. Furthermore, we found that ethical responsibility is indirectly associated with OCB through multiple pathways. Specifically, it is positively linked to OCB through ethical leadership and ethical climate (e.g., paths CSR → EC → OCB, CSR → EL → OCB, CSR → EC → MI → OCB, CSR → EL → EC → OCB, and CSR → EL → EC → MI → OCB). Conversely, it shows a negative link to OCB through ethical identity (e.g., paths CSR → MI → OCB and CSR → EL → MI → OCB). Our analysis highlights the important connection between ethical factors and OCB and provides suggestions for future research.

## Data Availability

The original contributions presented in the study are included in the article/Supplementary material, further inquiries can be directed to the corresponding author.

## References

[ref1] Anonymous (2021). The value of evidence synthesis. Nat. Hum. Behav. 5, 539–539. doi: 10.1038/s41562-021-01131-7, 34017128

[ref2] Aquino K. Reed A. (2002). The self-importance of moral identity. J. Pers. Soc. Psychol. 83, 1423–1440. doi: 10.1037/0022-3514.83.6.1423, 12500822

[ref3] Ashforth B. E. Mael F. (1989). Social identity theory and the organization. Acad. Manag. Rev. 14, 20–39. doi: 10.2307/258189

[ref4] Bandura A. (1977). Social Learning Theory. Englewood Cliffs: Prentice-Hall.

[ref5] Bandura A. (1986). Social Foundations of Thought and Action: A Social Cognitive Theory. Englewood Cliffs: Prentice-Hall, Inc.

[ref6] Barends E. Rousseau M. (2018). Evidence-Based Management—How to Use Evidence to Make Better Organizational Decisions. London: Kogan Page.

[ref7] Barnard C. I. (1968). The Functions of the Executive. Cambridge, MA: Harvard University Press.

[ref8] Bilgili T. Calderon C. Allen D. Kedia B. (2017). Gone with the wind: a meta-analytic review of executive turnover, its antecedents, and post acquisition performance. J. Manag. 43, 1966–1997. doi: 10.1177/0149206316635252

[ref9] Blau P. M. (1964). Exchange and Power in Social Life. New York: John Wiley & Sons.

[ref10] Caldwell C. (2011). Duties owed to organizational citizens – ethical insights for today’s leader. J. Bus. Ethics 102, 343–356. doi: 10.1007/s10551-011-0819-8

[ref11] Card N. A. (2011). Applied Meta-Analysis for Social Science Research. New York: Guilford Publications.

[ref12] Chen M. Chen C. Sheldon O. (2016). Relaxing moral reasoning to win: how organizational identification relates to unethical pro-organizational behavior. J. Appl. Psychol. 101, 1082–1096. doi: 10.1037/apl0000111, 27100068

[ref13] Cropanzano R. Mitchell M. S. (2005). Social exchange theory: an interdisciplinary review. J. Manag. 31, 874–900. doi: 10.1177/0149206305279602

[ref14] Den Hartog D. Belschak F. (2012). Work engagement and Machiavellianism in the ethical leadership process. J. Bus. Ethics 107, 35–47. doi: 10.1007/s10551-012-1296-4

[ref15] Deng C. (2020). The impact of Transformational Leadership on Teachers’ Organizational Citizenship Behavior: the Mediation effect of school Ethical Atmosphere (master’s thesis. Shanghai: East China Normal University.

[ref16] Ellemers N. De Gilder D. Haslam S. A. (2004). Motivating individuals and groups at work: a social identity perspective on leadership and group performance. Acad. Manag. Rev. 29, 459–478. doi: 10.5465/AMR.2004.13670967

[ref17] Estiri M. Amiri N. Khajeheian D. Rayej H. (2018). Leader-member exchange and organizational citizenship behavior in hospitality industry: a study on effect of gender. Eurasian Bus. Rev. 8, 267–284. doi: 10.1007/s40821-017-0083-7

[ref18] Gond J. P. El Akremi A. Swaen V. Babu N. (2017). The psychological. Microfoundations of corporate social responsibility: a person-centric systematic review. J. Organ. Behav. 38, 225–246. doi: 10.1002/job.2170

[ref19] Gong W. Li Z. Tang J. (2026). Impact of risk perception on emergency information seeking behavior: a meta-analysis. Front. Psychol. 16:1646584. doi: 10.3389/fpsyg.2025.1646584, 41583740 PMC12827699

[ref20] Hackett R. Wang A. Chen Z. Cheng B. Farh J. (2018). Transformational leadership and organizational citizenship behavior: a moderated mediation model of leader-member-exchange and subordinates’ gender. Appl. Psychol. 67, 617–644. doi: 10.1111/apps.12146

[ref21] Hung Y. Tsai Y. (2015). Ethical work climate and organizational citizenship behavior in the Taiwanese military. Mil. Psychol. 28, 34–49. doi: 10.1037/mil0000096

[ref22] Hunter E. Schmidt L. (2004). Methods of Meta-Analysis: Correcting Error and Bias in Research Findings. 2nd Edn Thousand Oaks: Sage.

[ref23] Kaur N. Kang L. S. (2023). The interplay of gendered identities and employees perception of organizational citizenship behavior. Evidence-based HRM: a Global Forum for Empirical Scholarship 11, 430–447. doi: 10.1108/EBHRM-05-2021-0106

[ref24] Kim J. (2018). The effects of relative organizational tenure on job Behaviors in the public sector. Public Pers. Manag 47, 335–355. doi: 10.1177/0091026017753646, 24564442

[ref25] LeBreton J. M. Senter J. L. (2008). Answers to 20 questions about interrater reliability and interrater agreement. Organ. Res. Methods 11, 815–852. doi: 10.1177/1094428106296642

[ref26] Lee K. Allen N. J. (2002). Organizational citizenship behavior and workplace deviance: the role of affect and cognitions. J. Appl. Psychol. 87, 131–142. doi: 10.1037/0021-9010.87.1.131, 11916207

[ref27] Li Z. Sha Y. Song X. Yang K. Zhao K. Jiang Z. . (2020). Impact of risk perception on customer purchase behavior: a meta-analysis. J. Bus. Ind. Mark. 35, 76–96. doi: 10.1108/JBIM-12-2018-0381

[ref28] Liao H. Allen T. D. Liu Z. Ptashnik T. Wu I. (2026). Uncovering a motherhood advantage: how parenthood impacts perceptions of the meaning of work and work outcomes. J. Appl. Psychol., online. doi: 10.1037/apl0001355, 41701276

[ref29] Liu Y. (2012). The impact mechanism of corporate citizenship behavior on employee organizational citizenship behavior: mediation model and moderating effect (Doctoral dissertation, Zhejiang University of Technology). doi: 10.7666/d.Y2297813

[ref30] Maignan I. Ferrell C. (2004). Corporate social responsibility and marketing: an integrative framework. J. Acad. Mark. Sci. 32, 3–19. doi: 10.1177/0092070303258971

[ref31] Mansur J. Sobral F. Islam G. (2020). Leading with moral courage: the interplay of guilt and courage on perceived ethical leadership and group organizational citizenship behaviors. Bus. Ethics: Eur. Rev. 29, 587–601. doi: 10.1111/beer.12270

[ref32] Mayer M. Aquino K. Greenbaum L. Kuenzi M. (2012). Who displays ethical leadership, and why does it matter? An examination of antecedents and consequences of ethical leadership. Acad. Manag. J. 55, 151–171. doi: 10.5465/amj.2008.0276

[ref33] Mcdaniel A. Rothstein R. Whetzel L. (2006). Publication bias: a case study of four test vendors. Pers. Psychol. 59, 927–953. doi: 10.1111/j.1744-6570.2006.00059.x

[ref34] Meyer P. Stanley J. Herscovitch L. Topolnytsky L. (2002). Affective, continuance, and normative commitment to the organization: a meta-analysis of antecedents, correlates, and consequences. J. Vocat. Behav. 61, 20–52. doi: 10.1006/jvbe.2001.1842

[ref35] Monin B. Miller T. (2001). Moral credentials and the expression of prejudice. J. Pers. Soc. Psychol. 81, 33–43. doi: 10.1037/0022-3514.81.1.33, 11474723

[ref36] Ogunfowora B. Nguyen Q. Steel P. Hwang C. (2022). A meta-analytic investigation of the antecedents, theoretical correlates, and consequences of moral disengagement at work. J. Appl. Psychol. 107, 746–775. doi: 10.1037/apl0000912, 34553966

[ref37] Oh S. Y. (2022). Effect of ethical climate in hotel companies on organizational trust and organizational citizenship behavior. Sustainability 14, 1–18. doi: 10.3390/su14137886, 30654563

[ref38] Organ W. (1988). Organizational Citizenship Behavior: The Good Soldier Syndrome. Lexington: Lexington Books.

[ref39] Organ W. (1997). Organizational citizenship behavior: it's construct clean-up time. Hum. Perform. 10, 85–97. doi: 10.1207/s15327043hup1002_2

[ref40] Organ W. Podsakoff M. MacKenzie B. (2006). Organizational Citizenship Behavior: Its Nature, Antecedents, and Consequences. Thousand Oaks, CA: Sage.

[ref41] Orlitzky M. Schmidt L. Rynes L. (2003). Corporate social and financial performance: a meta-analysis. Organ. Stud. 24, 403–441. doi: 10.1057/9780230594708_5

[ref42] Podsakoff P. M. MacKenzie S. B. Paine J. B. Bachrach D. G. (2000). Organizational citizenship behaviors: a critical review of the theoretical and empirical literature and suggestions for future research. J. Manag. 26, 513–563. doi: 10.1037/a0013079

[ref43] Podsakoff N. P. Whiting S. W. Podsakoff P. M. Blume B. D. (2009). Individual- and organizational-level consequences of organizational citizenship behaviors: a meta-analysis. J. Appl. Psychol. 94, 122–141. doi: 10.1037/a0013079, 19186900

[ref44] Qaiser D. Zhang L. (2023). Unveiling the mechanisms through which leader integrity shapes ethical leadership behavior: theory of planned behavior perspective. Behav. Sci. 13:928. doi: 10.3390/bs13110928, 37998675 PMC10669232

[ref45] Ruiz-Palomino P. Martínez-Baños R. (2014). Ethical culture, ethical intent, and organizational. Citizenship behavior: the moderating and mediating role of person–organization fit. J. Bus. Ethics 120, 95–108. doi: 10.1007/s10551-013-1650-1

[ref46] Saputra N. Putera R. E. Zetra A. Azwar A. Valentina T. R. Mulia R. A. (2026). Enhancing public management through integrity and organizational citizenship behavior: a systematic review. Int. J. Public Adm. 1-16, 1–16. doi: 10.1080/01900692.2026.2614578, 37339054

[ref47] Schmidt F. Le H. (2004). Software for the Hunter–Schmidt Meta-Analysis Methods. Iowa City: Department of Management and Organizations, University of Iowa.

[ref48] Shahzad K. Gu J. Mitchell R. Hong Y. De Sisto M. Luo Y. (2025). How and when ethics-oriented human resource management systems promote organizational citizenship behavior: the moderated mediation of work-family balance and moral attentiveness. Bus. Ethics Q. 35, 440–475. doi: 10.1017/beq.2024.23

[ref49] Shin Y. (2012). CEO ethical leadership, ethical climate, climate strength, and collective organizational citizenship behavior. J. Bus. Ethics 108, 299–312. doi: 10.1007/s10551-011-1091-7

[ref50] Syamsir S. Saputra N. Mulia R. A. (2026). Organizational citizenship behavior model in improving public service management mediated by integrity. Public Integrity, 1–25. doi: 10.1080/10999922.2026.2638672, 37339054

[ref51] Tang J. Li Z. (2026). The antecedents of organ donation intention and behavior: a meta-analysis. Health Commun. 41, 601–636. doi: 10.1080/10410236.2025.2522372, 40631352

[ref52] Trevino K. Weaver R. (2001). Organizational justice and ethics program “follow-through”: influences on employees' harmful and helpful behavior. Bus. Ethics Q. 11, 651–671. doi: 10.2307/3857765

[ref53] Turker D. (2009a). Measuring corporate social responsibility: a scale development study. J. Bus. Ethics 85, 411–427. doi: 10.1007/s10551-008-9780-6

[ref54] Turker D. (2009b). How corporate social responsibility influences organizational commitment. J. Bus. Ethics 89, 189–204. doi: 10.1007/s10551-008-9993-8

[ref55] Viswesvaran C. Ones D. S. (1995). Theory testing: combining psychometric meta-analysis and structural equations modeling. Pers. Psychol. 48, 865–885. doi: 10.1111/j.1744-6570.1995.tb01784.x

[ref56] Wang X. Jin J. Xu J. Khan M. (2025). Are women more likely to engage in extra green behaviors in the workplace? Gender differences in the spillover effect from employee in-role to extra-role green behavior. Front. Psychol. 16:1516658. doi: 10.3389/fpsyg.2025.1516658, 40357472 PMC12066582

[ref57] Wang Y. Sung W. (2014). Predictors of organizational citizenship behavior: ethical leadership and workplace jealousy. J. Bus. Ethics 135, 117–128. doi: 10.1007/s10551-014-2480-5, 30311153

[ref58] Wang B. Wang K. Y. (2018). Mechanisms of ethical management on organizational citizenship behavior. Bus. Manag. 2, 35–37. doi: 10.16517/j.cnki.cn12-1034/f.2018.02.011

[ref59] Wang J. Zheng W. Alam M. Murad M. Gul F. Gill S. A. (2022). The impact of transformational leadership on affective organizational commitment and job performance: the mediating role of employee engagement. Front. Psychol. 13:831060. doi: 10.3389/fpsyg.2022.831060 35465551 PMC9019157

[ref60] Williams L. J. Anderson S. E. (1991). Job satisfaction and organizational commitment as predictors of organizational citizenship and in-role behaviors. J. Manag. 17, 601–617. doi: 10.1177/014920639101700305, 18674448

[ref61] Xu L. Wen T. Wang J. (2022). How does job insecurity cause unethical pro-organizational behavior? The mediating role of impression management motivation and the moderating role of organizational identification. Front. Psychol. 13:941650. doi: 10.3389/fpsyg.2022.941650, 36211942 PMC9537742

[ref62] Yam K. C. Klotz A. C. He W. Reynolds S. J. (2017). From good soldiers to psychologically entitled: examining when and why citizenship behavior leads to deviance. Acad. Manag. J. 60, 373–396. doi: 10.5465/amj.2014.0234

[ref63] Yu X. Chen W. (2015). Research on the impact of organizational ethics climate on employee workplace behavior - using work dissociation as a mediating variable. J. Dalian Univ. Technol. (Soc. Sci.) 4, 35–40. doi: 10.19525/j.issn1008-407x.2015.04.006

[ref64] Zhang G. Zhu Y. (2020). Moral leadership and organizational citizenship behavior: an interactive perspective of social learning and attribution theory. Technol. Econ 33, 76–80. doi: 10.14059/j.cnki.cn32-1276n.2020.02.016

