# Supplementary material for: Do ethics matter? Impact of ethics on organizational citizenship behavior: a meta-analysis
Source: Front Psychol. 2026 May 19;17:1773853. doi: 10.3389/fpsyg.2026.1773853 (PMC13225955; doi:10.3389/fpsyg.2026.1773853)
Supplement: Supplementary file 1 [file Supplementary_File_1.DOCX]

**Supplementary material**

**Effect size computation**

We assessed studies that used a correlation coefficient to describe the relationship between risk perception and customer purchase behavior. The correlation coefficient was typically expressed in $r$. However, a small number of studies reported other coefficients, such as $d$, SE, and $R^{2}$. In such cases, it was necessary to convert these coefficients into $r$, as follows (Card, 2011)^33^:

$$r=\frac{d}{\sqrt{4+d}}$$

$d=\frac{m1-m2}{s}$, m1 and m2 were the means of group 1 and group 2, and $s$ was the pooled standard deviation across the two groups.

$$r=\frac{{(1-R^{2})}^{2}}{n-1}$$

$R^{2}$ was the determinant coefficient, and $n$ was the sample size.

$Z_{i}=0.5ln(\frac{1+r_{i}}{r_{i}})$, ${SE}_{i}=\frac{1}{\sqrt{W_{i}}}$, $Z=\frac{\sum(W_{i}* Z_{i})}{\sum W_{i}}$, $r=\frac{e^{2z}-1}{e^{2z}+1}$

$W_{i}$= n – 3, where Z was the Z score when calculating Fisher's Z.

**Overall effects value**

**Table A1** The overall effects value meta-analysis.

| **Independent variable** | **Dependent variable** | **Model** | **k** | **N** | ρ | **Lower**  **95%CI** | **Upper 95%CI** | **Z-value** |
| --- | --- | --- | --- | --- | --- | --- | --- | --- |
| 28 ethical factors | OCB | Fixed | 125 | 158,336 | 0.264*** | 0.261 | 0.266 | 191.403 |
|  |  | Random | 125 | 158,336 | 0.286*** | 0.244 | 0.326 | 12.186 |

**Notes:** *, * *, and * * * represents p<0.1, p<0.05, and p<0.01.

**Table A2** The results of robustness checks.

| **Independent variable** | **Dependent variable** | **Methods** | **Model** | **k** | **N** | ρ | **Lower**  **95%CI** | **Upper 95%CI** | **Z-value** |
| --- | --- | --- | --- | --- | --- | --- | --- | --- | --- |
| 28 ethical factors | OCB | One study removed | Random d | 125 | 158,336 | 0.286*** | 0.244 | 0.326 | 12.186 |
|  |  | cumulative analysis | Random | 125 | 158,336 | 0.286*** | 0.244 | 0.326 | 12.186 |

**Notes:** *, * *, and * * * represents p<0.1, p<0.05, and p<0.01.

**
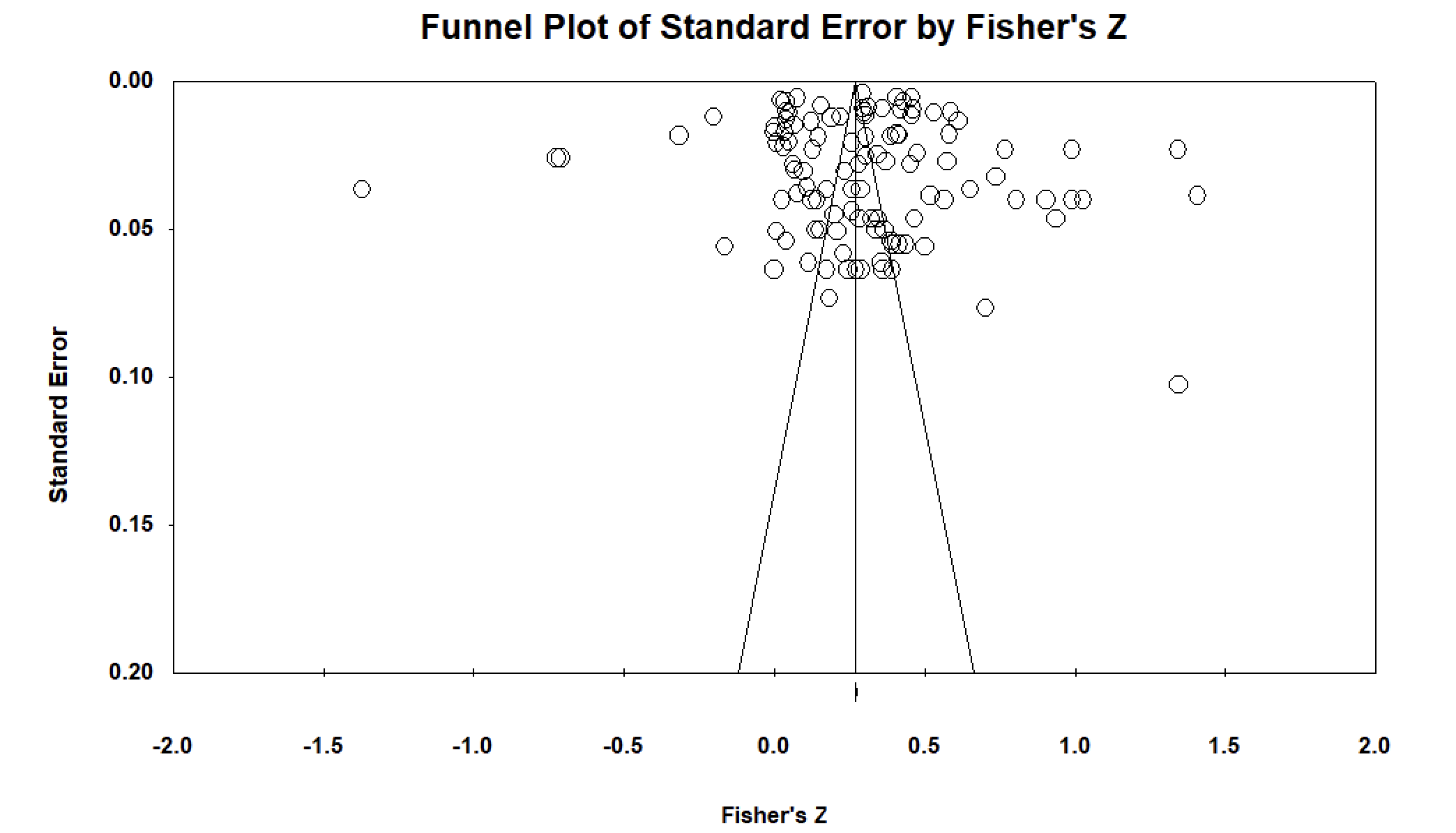
**

**Figure A1** The overall funnel plot of 125 articles.

**Studies Included in this study**

Abdelmotaleb, M., & Saha, S. K. (2019). Corporate social responsibility, public service motivation and organizational citizenship behavior in the public sector. *International Journal of Public Administration*, 42(11), 929-939.

Abdulkareem, R., & Tarik, A. (2018). The influence of ethical leadership on academic employees' organizational citizenship behavior and turnover intention. *Management Decision*, 57(3), 583-605.

Abu B., & Connaughton, S. (2022). Ethical leadership, perceived leader–member ethical communication and organizational citizenship behavior: development and validation of a multilevel model. *Leadership & Organization Development Journal*, 43(1), 96-110.

Alhyasat, K. (2012). The role of Islamic work ethics in developing organizational citizenship behavior at the Jordanian Press Foundations. *Journal of Islamic Marketing*, 3, 139-154.

Ali, H., Yin, J., Manzoor, F., & An, M. (2023). The impact of corporate social responsibility on firm reputation and organizational citizenship behavior: The mediation of organic organizational cultures. *Frontiers in Psychology*, 13, 1100448, 1-16.

Aloustani, S., Atashzadeh-Shoorideh, F., Zagheri-Tafreshi, M., Nasiri, M., Barkhordari-Sharifabad, M., & Skerrett, V. (2020). Association between ethical leadership, ethical climate and organizational citizenship behavior from nurses' perspective: a descriptive correlational study. *BMC nursing*, 19(15), 1-8.

Alshihabat, K., & Atan, T. (2020). The mediating effect of organizational citizenship behavior in the relationship between transformational leadership and corporate social responsibility practices: middle eastern example/jordan. *Sustainability*, 12, 4248, 1-17.

Altunta, S., Ntepeler, S., Skmen, S., Kantek, Ztürk, F., & Baykal. (2020). The effect of ethical work climate on the organizational citizenship behavior of academic nurses. International Nursing Review, 15-23.

and organizational citizenship behaviour-The mediating role of job satisfaction. Journal of Global Responsibility, 10, 47-68.

Arokiasamy, A., Maheshwari, G., & Nguyen, K. (2022). The Influence of Ethical and Transformational Leadership on Employee Creativity in Malaysia's Private Higher Education Institutions: The Mediating Role of Organizational Citizenship Behaviour. 17. 1-33.

Arshad, M., Abid, G., & Torres, F. V. C. (2021). Impact of prosocial motivation on organizational citizenship behavior: the mediating role of ethical leadership and leader–member exchange. *Quality & Quantity: International Journal of Methodology*, 55, 133-150.

Bai, S., &An, L. (2014). Empirical study on the impact of perceived corporate ethics on employees' attitudes and behaviors. *Ethics Research*, (05), 128-134.

Baker, T. L., Hunt, T. G., & Andrews, M. (2010). Promoting ethical behavior and organizational citizenship behaviors: the influence of corporate ethical values. *Social Science Electronic Publishing*, 59(7), 849-857.

Boan, E., & Dedeolu, B. B. (2020). Hotel employees' corporate social responsibility perception and organizational citizenship behavior: perceived external prestige and pride in organization as serial mediators. *Corporate Social Responsibility and Environmental Management*, 27, 2343-2353.

Bonner, J. M., Greenbaum, R. L., & Mayer, D. M. (2016). My boss is morally disengaged: the role of ethical leadership in explaining the interactive effect of supervisor and employee moral disengagement on employee behaviors. *Journal of Business Ethics*, 137(4), 731-742.

Castro-Gonzalez, S., Bande, B., & Kimura, T. (2019). How and when corporate social responsibility affects salespeople's organizational citizenship behaviors?: the moderating role of ethics and justice. *Corporate Social Responsibility and Environmental Management*, 26(3), 548-558.

Chen, C., Chou, Y., & Lee, C. (2021). Social Innovation, Employee Value Cocreation, and Organizational Citizenship Behavior in a Sport-Related Social Enterprise: Mediating Effect of Corporate Social Responsibility. *Sustainability*, 13,12582, 1-10.

Chen, D., & Liu, B. (2021). Empirical Study on the Impact of Ethical Leadership on the Change Oriented Organizational Citizenship Behavior of Grassroots Civil Servants. *Journal of Central South University (Social Sciences Edition)*, (03), 100-111.

Chen, H. (2021). Research on the Impact of Ethical Leadership and Subordinate Relations on Organizational Citizenship Behavior (Master's Thesis, Yunnan University of Finance and Economics). https://kns.cnki.net/KCMS/detail/detail.aspx?dbname=CMFD202102&filename=1021614988.nh

Chen, X. (2015). Empirical Study on the Impact of Ethical Leadership on Organizational Citizenship Behavior (Master's Thesis, Northeastern University of Finance and Economics). https://kns.cnki.net/KCMS/detail/detail.aspx?dbname=CMFD201502&filename=1015550150.nh

Chen, X., Hansen, E., & Cai, J. (2022). Synthesizing and comparing the different effects between internal and external corporate social responsibility perceptions and organizational citizenship behavior: A need theory perspective. *Corporate Social Responsibility and Environmental Management*, 1-14.

Choi, J., Sohn, Y. W., & Lee, S. (2020). The effect of corporate social responsibility on employees' organizational citizenship behavior: a moderated mediation model of grit and meaning orientation. *Sustainability*, 12, 54111, 1-14.

Chun, J. S., Shin, Y., Choi, J. N., & Kim, M. S. (2013). How does corporate ethics contribute to firm financial performance? the mediating role of collective organizational commitment and organizational citizenship behavior. *Journal of Management*, 39(4), 853-877.

Cingöz, A., & Asuman, A. (2019). A study on determining the relationships among corporate social responsibility, organizational citizenship behavior and ethical leadership. *International Journal of Innovation and Technology Management (IJITM)*, 16, 1-19.

Cui, J. (2015). Research on the correlation between moral practice ability, organizational identity, and organizational citizenship behavior (Master's thesis, Chongqing University). https://kns.cnki.net/KCMS/detail/detail.aspx?dbname=CMFD201601&filename=1015966487.nh

Dany, F., & Zied, G. (2008). The international journal of human resource management. *The International* *Journal of Human Resource Management*, 19(11), 2095-2112.

Deng, C. (2016). Research on the Relationship between Moral Leadership, Workplace Spirituality, and Organizational Citizenship Behavior (Master's Thesis, Northeastern University of Finance and Economics). https://kns.cnki.net/KCMS/detail/detail.aspx?dbname=CMFD201701&filename=1017049153.nh

Deng, C. (2020). The impact of transformational leadership on teachers' organizational citizenship behavior: the mesomeric effect of school ethical atmosphere (master's thesis, East China Normal University). https://kns.cnki.net/KCMS/detail/detail.aspx?dbname=CMFD202002&filename=1020635899.nh

Du, Y. (2015). The impact of ethical leadership on employee behavior - a test of the mesomeric effect (master's thesis, Guangxi University of Science and Technology). https://kns.cnki.net/KCMS/detail/detail.aspx?dbname=CMFD201702&filename=1016713544.nh

Fan, H., & Zhou, Z. (2017). Research on the Impact of Ethical Leadership on Team Autonomous Behavior - Based on the Perspective of Team Moral Culture. *Soft science*, (10), 71-75.

Fan, H., & Zhou, Z. (2018). Ethical Leadership and Employee Autonomous Behavior: From the Perspective of Social Learning Theory. *Management Review*, (09), 164-173.

Farid, T., Iqbal, S., Ma, J., Tayyiba, M., & Mehmood, Q. (2017). Effect of Islamic work ethics on employees work engagement and organizational citizenship behavior. *Psychologia*, 60, 111-120.

Fida, R., Paciello, M., Tramontano, C., Fontaine, R. G., Barbaranelli, C., & Farnese, M. L. (2015). An integrative approach to understanding counterproductive work behavior: the roles of stressors, negative emotions, and moral disengagement. *Journal of Business Ethics*, 130(1), 131-144.

Freire, Ca., Gonçalves, J., & Carvalho, M. (2022). Corporate Social Responsibility: The Impact of Employees’ Perceptions on Organizational Citizenship Behavior through Organizational Identification. *Administrative Sciences*, 12, 120, 1-18.

Gao, Y. (2019). The Relationship between Perception of Psychological Contract Breach and Organizational Citizenship Behavior - The Role of Work Ethics and Types of Psychological Contract (Master's Thesis, Zhejiang University). https://kns.cnki.net/KCMS/detail/detail.aspx?dbname=CMFD202001&filename=1019246509.nh

Geng, Q. (2016). A Study on the Dual Dimensional Pathway Influencing Organizational Citizenship Behavior of Primary and Secondary School Teachers (Master's Thesis, Jiangnan University). https://kns.cnki.net/KCMS/detail/detail.aspx?dbname=CMFD201701&filename=1016265200.nh

Gerpott, F. H., Quaquebeke, N. V., Schlamp, S., & Voelpel, S. C. (2019). An identity perspective on ethical leadership to explain organizational citizenship behavior: the interplay of follower moral identity and leader group prototypicality. *Journal of Business Ethics*, 156, 1063-1078.

Ghosh, K., Humphreys, P., & Humphreys, P. (2002). Benevolent leadership in not-for-profit organizations: welfare orientation measures, ethical climate and organizational citizenship behaviour. *Journal of International Business Studies*, 33(3), 629-632.

Griep, Y., Germeys, L., & Kraak, J. (2021). Unpacking the relationship between organizational citizenship behavior and counterproductive work behavior: moral licensing and temporal focus:. *Group & Organization Management*, 46(5), 819-856.

Guan, R. (2021). Corporate hypocrisy and employee organizational citizenship behavior (Master's thesis, Henan University). https://kns.cnki.net/KCMS/detail/detail.aspx?dbname=CMFD202202&filename=1021776931.nh

Guo, S. (2021). The Impact of the Ethical Atmosphere of Young Pioneers' Caring Organizations on the Organizational Citizenship Behavior of Young Pioneers in Middle and High Levels of Primary School (Master's Thesis, Shihezi University). https://kns.cnki.net/KCMS/detail/detail.aspx?dbname=CMFD202201&filename=1021838959.nh

Guo, S., & Xiao, M. (2017). The Impact of Moral Characteristics and Moral Identity on the Moral Behavior of State owned Enterprise Employees: Differences in Action and Interaction Effects. *Journal of Northeast University (Social Sciences Edition)*, (05), 476-482.

Haider, S., Sarwar, N., Akhtar, M., & Khuda, B. (2022). Moderated-mediation between ethical leadership and organizational citizenship behavior: the role of psychological empowerment and high performance managerial practices. *Management Research Review*, 46.

He, X. (2011). Research on the impact and mechanism of corporate social responsibility and ethical leadership behavior on employee organizational citizenship behavior (doctoral thesis, Southwest Jiaotong University). https://kns.cnki.net/KCMS/detail/detail.aspx?dbname=CDFD1214&filename=1012389626.nh

Huang, N., Qiu, S., Yang, S., & Deng, R. (2021). Ethical leadership and organizational citizenship behavior: mediation of trust and psychological well-being. *Psychology Research and Behavior Management*, 14, 655-664.

Hung, Y. C., & Tsai, T. Y. (2015). Ethical work climate and organizational citizenship behavior in the taiwanese military. *Military Psychology*, 28, 34-49.

Iqbal, S., Farid, T., Ma, J., Khattak, A., & Nurunnabi, M. (2018). The Impact of Authentic Leadership on Organizational Citizenship Behaviours and the Mediating Role of Corporate Social Responsibility in the Banking Sector of Pakistan. *Sustainability*, 10, 2170, 1-11.

Jiang, W., Liang, B., & Wang, L. (2022). The double-edged sword effect of unethical pro-organizational behavior: the relationship between unethical pro-organizational behavior, organizational citizenship behavior, and work effort. *Journal of Business Ethics*, 183(4), 1159-1172.

Khan, M. A. S., Du, J. G., Ali, M., Saleem, S., & Usman, M. (2019). Interrelations between ethical leadership, green psychological climate, and organizational environmental citizenship behavior: a moderated mediation model. *Frontiers in Psychology*, 10, 1977.

Lee, S., & Ha-Brookshire, J. (2018). The effect of ethical climate and employees' organizational citizenship behavior on U.S. fashion retail organizations' sustainability performance. *Corporate Social Responsibility and Environmental Management*, 25, 939-947.

Liang, H., Li, X., & Chen, B. (2019). Will ethical leadership turnover necessarily affect employee organizational citizenship behaviour-A mediating regulatory model. *Prediction*, (04), 1-9.

Liu, F. (2009). Empirical Study on the Relationship between Ethical Climate, Organizational Commitment, and Organizational Citizenship Behavior of Private Enterprises in Wenzhou. *Proceedings of the Theoretical Seminar on "Order and Progress: A 60 Year Study of Zhejiang Social Development" and the 2009 Zhejiang Sociological Annual Conference*, 342-355.

Liu, F. (2010). The relationship between corporate ethical climate, organizational commitment, and organizational citizenship behavior: A case study of private enterprises in Wenzhou. *Journal of Shenyang University of Technology (Social Sciences Edition)*, (04), 366-374.

Liu, F., Li, J., & Yang, L. (2017). Research on the Relationship between Corporate Social Responsibility, Moral Identity, and Employee Organizational Citizenship Behavior. *China soft science,* (06), 117-129.

Liu, T. (2018). Research on the Process of the Role of Citizen Behavior in Constructive Deviation Behavior: From the Perspective of Moral Permission (Doctoral Dissertation, Huazhong University of Science and Technology). https://kns.cnki.net/KCMS/detail/detail.aspx?dbname=CDFDLAST2019&filename=1019819172.nh

Liu, Y. (2012). Exploring the Mechanism of the Effect of Corporate Ethics on Organizational Citizenship Behavior. *The Business Era*, (08), 95-97.

Zúñiga, C., Aguado, D., & Cabrera-Tenecela, P. (2022). Values that work: Exploring the moderator role of protestant work ethics in the relationship between human resources practices and work engagement and organizational citizenship behavior. *Administrative Sciences*, 12(1), 11.

Lu, X. (2014). Ethical leadership and organizational citizenship behavior: the mediating roles of cognitive and affective trust. *Social Behavior and Personality: an international journal*, 42(8), 1365-1376.

Raza, S. A., Khan, K. A., & Hakim, F. (2024). Whether organizational citizenship behavior is triggered by employee CSR perception and spiritual values: the moderating role of Islamic work ethics. *Management Research Review*, 47(3), 353-373.

Malik, S. Y., Mughal, Y. H., Azam, T., Cao, Y., Wan, Z., & Zhu, H. (2021). Corporate social responsibility, green human resources management, and sustainable performance: is organizational citizenship behavior towards environment the missing link?. *Sustainability*, 13, 1044, 1-24.

Mansur, J., Sobral, F., & Islam, G. (2020). Leading with moral courage: the interplay of guilt and courage on perceived ethical leadership and group organizational citizenship behaviors. *Business Ethics: A European Review*, 29, 587-601.

Matherne, C. F., Ring, J. K., & Farmer, S. (2018). Organizational moral identity centrality: relationships with citizenship behaviors and unethical prosocial behaviors. *Journal of Business and Psychology*, 33, 711-726.

Mithulan, R., & Opatha, H. (2023). The Moderating Effect of Personal Character and Mediating Effect of Organizational Citizenship Behavior on Ethical Orientation of HRM-Ethical Behavior Linkage. *Sri Lankan Journal of Human Resource Management*, 13, 1-26.

Mo, S., & Shi, J. (2017). Linking ethical leadership to employees' organizational citizenship behavior: testing the multilevel mediation role of organizational concern. *Journal of Business Ethics*, 141(1), 151-162.

Mohammad, J., Quoquab, F., & Omar, R. (2016). Factors Affecting Organizational Citizenship Behavior among Malaysian Bank Employees: The Moderating Role of Islamic Work Ethic. Procedia - Social and Behavioral Sciences, 224, 562-570.

Shahzad, K., Gu, J., Mitchell, R., Hong, Y., De Sisto, M., & Luo, Y. (2025). How and When Ethics-oriented Human Resource Management Systems Promote Organizational Citizenship Behavior: The Moderated Mediation of Work-Family Balance and Moral Attentiveness. *Business Ethics Quarterly*, 1-36.

Mostafa, A. . (2018). Ethical leadership and organizational citizenship behaviours: the moderating role of organizational identification. *European Journal of Work and Organizational Psychology*, 27(4), 1-9.

Mu, H. (2022). The impact of personal career management on organizational citizenship behavior among new generation employees: a moderated mediation model (Master's thesis, Qufu Normal University). https://kns.cnki.net/KCMS/detail/detail.aspx?dbname=CMFDTEMP&filename=1022032290.nh

Murtaza, G., Abbas, M., Usman, b., Raja, U., Roques, O., Afsheen, b., bullet, K., & Mushtaq, R. (2014). Impact of Islamic Work Ethics on Organizational Citizenship Behaviors and Knowledge-Sharing Behaviors. *Journal of Business Ethics*, 133, 325-333.

Nemr, M. A. A., & Liu, Y. (2021). The impact of ethical leadership on organizational citizenship behaviors: moderating role of organizational cynicism. *Cogent Business & Management*, 8(1), 1865860, 1-15.

Nurcholis, L., & Palupi, S. (2022). Peningkatan komitmen afektif berbasis Islamic work ethics, spiritual leadership dan perceived organizational support terhadap organizational citizenship behavior. *Jurnal Riset Ekonomi dan Bisnis*, 15, 234-254.

Oh, S. Y. (2022). Effect of ethical climate in hotel companies on organizational trust and organizational citizenship behavior. *Sustainability*, 14, 7886, 1-18.

Ouakouak, M. L., Arya, B., & Zaitouni, M. (2020). Corporate social responsibility and intention to quit mediating role of organizational citizenship behavior. *International journal of productivity and performance management*, (3), 69, 447-465.

Peng, T., Yap, C., Choong, Y., & Choe, K., & Rungruang, P., & Li, Zhen. (2019). Ethical leadership, perceived organizational support and citizenship behaviors: The moderating role of ethnic dissimilarity. *Leadership & Organization Development Journal*, 40(8), 877-897.

Pio, R., & Lengkong, F. (2020). The relationship between spiritual leadership to quality of work life and ethical behavior and its implication to increasing the organizational citizenship behavior. *Journal of Management Development*, 39(3), 293-305.

Tran, N. K. H. (2025). The impact of corporate environment ethics and mediating role of organizational identification and employee environmental commitment on environmental citizenship behaviors. *Journal of Fashion Marketing and Management: An International Journal*, 29(5), 915-934.

Qiu, S., Dooley, L. M., Deng, R., & Li, L. (2020). Does ethical leadership boost nurses' patient-oriented organizational citizenship behaviors? a cross-sectional study. *Journal of Advanced Nursing*, 76(2), 1603-1613.

Ruiz-Palomino, P., & Martinez-Canas, R. (2014). Ethical culture, ethical intent, and organizational citizenship behavior: the moderating and mediating role of person–organization fit. *Journal of Business Ethics*, 120(1), 95-108.

Ruiz-Palomino, P., Ruiz-Amaya, C., & Knorr, H. (2011). Employee organizational citizenship behaviour: the direct and indirect impact of ethical leadership. *Revue Canadienne des Sciences de l'Administration-Canadian Journal of Administrative Sciences*, 28(3), 244-244-258.

Ryan, J. J. (2001). Moral reasoning as a determinant of organizational citizenship behaviors: a study in the public accounting profession. *Journal of Business Ethics*, 33(3), 233-244.

Seth, M., Sethi, D., Kumar L., & Malik, N. (2022). Is ethical leadership accentuated by perceived justice?: Communicating its relationship with organizational citizenship behavior and turnover intention. *Corporate Communications: An International Journal*, 27(10), 705-723.

Sharma, D. (2018). When fairness is not enough: impact of corporate ethical values on organizational citizenship behaviors and worker alienation. *Journal of Business Ethics*, 150(1), 1-12.

Shi, W. (2016). Research on the Impact of Organizational Moral Climate on Organizational Citizenship Behavior (Master's Thesis, Nanjing University of Aeronautics and Astronautics). https://kns.cnki.net/KCMS/detail/detail.aspx?dbname=CMFD201701&filename=1016791376.nh

Song, B. (2019). Research on the Impact of Organizational Moral Climate on Organizational Citizenship Behavior (Master's Thesis, Guilin University of Electronic Science and Technology). https://kns.cnki.net/KCMS/detail/detail.aspx?dbname=CMFD202001&filename=1019919374.nh

Suryani, S., Sudrajat, B., Hendryadi, H., Saihu, M., Amalia, E., & Fathoni, M. (2022). Development of thriving at work and organizational citizenship behavior through Islamic work ethics and humble leadership. *Asian Journal of Business Ethics*, 1-23.

Tahir, F. (2018). The impact of organizational fairness and Islamic work ethics on work engagement and organizational citizenship behavior (doctoral thesis, Zhejiang University). https://kns.cnki.net/KCMS/detail/detail.aspx?dbname=CDFDLAST2022&filename=1019005128.nh

Tang, L. (2017). Research on the Impact of Ethical Leadership on Employee Organizational Citizenship Behavior (Master's Thesis, Guangdong University of Finance and Economics). https://kns.cnki.net/KCMS/detail/detail.aspx?dbname=CMFD201801&filename=1017851999.nh

Teng, C., Lu, A., Huang, Z., & Fang, C. (2019). Ethical work climate, organizational identification, leader-member-exchange (LMX) and organizational citizenship behavior (OCB): A study of three star hotels in Taiwan. *International Journal of Contemporary Hospitality Management*, 32(1), 212-229.

Tian, J. (2015). Research on the Impact of Moral Leadership on Organizational Citizenship Behavior (Master's Thesis, Harbin Institute of Technology). https://kns.cnki.net/KCMS/detail/detail.aspx?dbname=CMFD201601&filename=1015982001.nh

Tuan, L. T., & Ngan, V. T. (2021). Leading ethically to shape service-oriented organizational citizenship behavior among tourism salespersons: dual mediation paths and moderating role of service role identity. *Journal of Retailing and Consumer Services*, 60, 102421.

Vridyaningtyas, E. (2022). PENGARUH ORGANIZATIONAL CITIZENSHIP BEHAVIOR (OCB) DAN KOMPENSASI TERHADAP KINERJA KARYAWAN DAN WORK OVERLOAD SEBAGAI VARIABEL MEDIASI PADA PT. NASMOCO BAHTERA MOTOR DI YOGYAKARTA. Jurnal Studi Manajemen Organisasi, 17, 19-26.

Wang, C. (2014). Exploration of the Relationship between Ethical Atmosphere of Caring Organizations and Organizational Citizenship Behavior. *The Business Era*, (03), 114-116.

Wang, F. (2020). Research on the Impact of Corporate Social Responsibility Matching on Organizational Citizenship Behavior (Master's Thesis, North China University of Technology). https://kns.cnki.net/KCMS/detail/detail.aspx?dbname=CMFD202101&filename=1020360771.nh

Wang, Gordon & Hackett, Rick. (2022). Virtuous leadership, moral behavior, happiness and organizational citizenship: the mediating effect of virtues-centered moral identity. *Leadership & Organization Development Journal*, 43(7), 1043-1062.

Wang, J. (2019). Research on the Impact of Civil Servant Integrity Values on. Organizational Citizenship Behavior (Master's Thesis, Lanzhou University) https://kns.cnki.net/KCMS/detail/detail.aspx?dbname=CMFD201902&filename=1019875939.nh

Wang, L., Dong, X., An, Y., Chen, C., Eckert, M., Sharplin, G., Fish, J., & Fan, X. (2022). Relationships between job burnout, ethical climate and organizational citizenship behavior among registered nurses: A cross‐sectional study. *International Journal of Nursing Practice*, e13115, 1-13.

Wang, L., Li, D., Wei, W., Zhang, T., Tang, W., & Lu, Q. (2022). The impact of clinical nurses' perception of hospital ethical climates on their organizational citizenship behavior: A cross-sectional questionnaire survey. *Medicine*, 101, e28684, 1-7.

Wang, R. (2017). Ethical leadership and subordinate work happiness and organizational citizenship behavior. *Chinese Journal of clinical psychology*, (05), 939-948.

Wang, Y. (2013). Empirical Study on Moral Leadership and Organizational Citizenship Behavior (Master's Thesis, Capital University of Economics and Trade). https://kns.cnki.net/KCMS/detail/detail.aspx?dbname=CMFD201401&filename=1013224767.nh

Wang, Y., & Sung, W. (2014). Predictors of Organizational Citizenship Behavior: Ethical Leadership and Workplace Jealousy. *Journal of Business Ethics*, 135, 1-12.

Wen, J., Chen, Z., & Du, X. (2020). The impact of ethical leadership. on the civic behavior of environmental organizations. *Modern Economic Information,* (01), 18-20.

Xi, H. (2009). Research on the Mechanism of the Characteristics of Corporate Social Responsibility on Employee Organizational Commitment and Organizational Citizenship Behavior (Doctoral Dissertation, Zhejiang University). https://kns.cnki.net/KCMS/detail/detail.aspx?dbname=CDFD0911&filename=2010038153.nh

Xie, H., Shi, J., Mo, S., &Xie, J. (2022). The impact of ethical leadership on employee service oriented organizational citizenship behaviour. *Management Science*, (05), 99-112.

Xing, Z., He, W., Zhang, Z., &Jiang, X. (2022). The impact of employee ethical leadership prototypes on the effectiveness of ethical leadership: the mediating role of employee reverence. *Journal of Psychology*, (09), 1093-1105.

Yang, C., Ding, C., & Lo, K. (2015). Ethical Leadership and Multidimensional Organizational Citizenship Behaviors: The Mediating Effects of Self-Efficacy, Respect, and Leader-Member Exchange. *Group & Organization Management*, 41, 343-374.

Yang, Q., & Wei, H. (2017). The impact of ethical leadership on organizational citizenship behavior: the moderating role of workplace ostracism. *Leadership & Organization Development Journal*, 39(1), 100-113.

Youn, H., & Kim, J (2022). Corporate Social Responsibility and Hotel Employees’ Organizational Citizenship Behavior: The Roles of Organizational Pride and Meaningfulness of Work. *Sustainability*, 14, 2428, 1-18.

Yu, X., & Chen, W. (2015). Research on the Impact of Organizational Ethics Climate on Employee Workplace Behavior - Using Work Dissociation as a Mediating Variable. *Journal of Dalian University of Technology (Social Sciences Edition).* (04), 35-40.

Yu, Z. (2013). A Study on the Mechanism of the Impact of Corporate Ethical Behavior on Employee Organizational Citizenship Behavior - A Mediated Model of moderating Variables. *Modern Business*, (32), 90-92.

Yu, Z. (2013). Research on the Mechanism of the Impact of Employee Oriented Corporate Ethical Behavior on Employee Organizational Citizenship Behavior (Master's Thesis, Zhejiang University). https://kns.cnki.net/KCMS/detail/detail.aspx?dbname=CMFD201401&filename=1014114233.nh

Yuhyung, S. (2012). CEO Ethical Leadership, Ethical Climate, Climate Strength, and Collective Organizational Citizenship Behavior. *Journal of Business Ethics*, 108(3), 299–312.

Yunanto, Y., Suhariadi, F., Yulianti, P., Pangastuti, R. L., & Yanuarita, H. A. (2021). The effect of ethical leadership on organizational citizenship behavior: an empirical study in indonesia. *Korea Distribution Science Association*, 8(7), 285-294.

Zhang, F. (2017). Empirical Study on the Impact of Ethical Leadership on Organizational Citizenship Behavior (Master's Thesis, Jilin University). https://kns.cnki.net/KCMS/detail/detail.aspx?dbname=CMFD201702&filename=1017158727.nh

Zhang, G., & Zhu, Y. (2020). Moral leadership and organizational citizenship behavior: an interactive perspective of social learning and attribution theory. *Technology and Economy*, (02), 76-80.

Zhang, R. (2011). Empirical Study on the Relationship between Employee Organizational Citizenship Behavior and Work Ethics (Master's Thesis, Shanxi University). https://kns.cnki.net/KCMS/detail/detail.aspx?dbname=CMFD2012&filename=1012279429.nh

Zhang, S. (2013). Empirical Study on the Impact of Organizational Moral Climate on Organizational Citizenship Behavior (Doctoral Dissertation, Central South University). https://kns.cnki.net/KCMS/detail/detail.aspx?dbname=CDFD1214&filename=1014403417.nh

Zhang, S., Li, M., & Yan, A. (2014). The Relationship between Organizational Moral Climate, Supervisor Trust, and Organizational Citizenship Behavior. *Journal of Management*, (01), 61-68.

Zhang, W. (2011). An empirical study on the mediating effect of organizational trust on the impact of moral leadership on organizational citizenship behavior. (Master's Thesis, Southwest Jiaotong University). https://kns.cnki.net/KCMS/detail/detail.aspx?dbname=CMFD201301&filename=1012391914.nh

Zhang, Z. (2019). Moral Leadership and Employee Organizational Citizenship Behavior: The Role of Organizational Fairness and Power Distance (Master's Thesis, Nanjing Normal University). https://kns.cnki.net/KCMS/detail/detail.aspx?dbname=CMFD202001&filename=1019256467.nh

Zhao, H., Zhou, Q., He, P., & Jiang, C. (2021). How and when does socially responsible hrm affect employees' organizational citizenship behaviors toward the environment?:. *Journal of Business Ethics*, 169(2), 371-385.

Zhao, J. (2014). Research on the antecedents and outcomes of employee moral equity (Master's thesis, Zhejiang University). https://kns.cnki.net/KCMS/detail/detail.aspx?dbname=CMFD201401&filename=1014167992.nh

Zhou, S. (2021). Research on the Impact of Ethical Leadership on Employee Environmental Protection Organizational Citizenship Behavior (Master's Thesis, Shandong University). https://kns.cnki.net/KCMS/detail/detail.aspx?dbname=CMFD202201&filename=1021030060.nh

Zhu, Y. (2019). Perceived Moral Leadership and Subordinate Organizational Citizenship Behavior (Master's Thesis, Zhejiang University of Technology). https://kns.cnki.net/KCMS/detail/detail.aspx?dbname=CMFD202101&filename=1020439821.nh

Zhu, Z. (2016). The Impact of Confucian Ethical Leadership on Enterprise Innovation (Master's Thesis, Nanjing University). https://kns.cnki.net/KCMS/detail/detail.aspx?dbname=CMFD202201&filename=1016136764.nh
